# Immune subversion by *Leishmania infantum* parasites suppresses NLRP3-driven inflammatory responses in amyloid-β-activated microglia

**DOI:** 10.1186/s12974-025-03574-5

**Published:** 2025-10-29

**Authors:** Estefanía Calvo Alvarez, Chiara Sfogliarini, Francesca La Rosa, Marina Saresella, Maria Dolci, Elisabetta Vegeto, Donatella Taramelli, Nicoletta Basilico, Mario Clerici

**Affiliations:** 1 Department of Pharmacological and Biomolecular Sciences, Laboratory of Parasitology, Molecular Virology and Immunology, University of Milan, Milan, Italy; 2 Department of Pharmaceutical Sciences, University of Milan, Milan, Italy; 3 Laboratory of Molecular Medicine and Biotechnology, IRCCS Fondazione Don Carlo Gnocchi, Milan, Italy; 4 Department of Biomedical, Surgical and Dental Sciences, Laboratory of Parasitology, Molecular Virology and Immunology, University of Milan, Milan, Italy; 5 Department of Pathophysiology and Transplantation, University of Milan, Milan, Italy

**Keywords:** Microglia, *Leishmania infantum*, Immune subversion, Amyloid-β, NLRP3 inflammasome, Innate immunity, Alzheimer’s disease, Microglia-mediated inflammatory responses

## Abstract

Chronic activation of innate immune responses in the brain is increasingly recognized as a contributor to neurodegenerative diseases, including Alzheimer's disease (AD). AD remains a major global health challenge due to the inefficacy of current therapies to modify disease progression. In AD, hyperactivated microglia, the brain’s resident macrophages, play a central role by responding to amyloid-beta peptides (Aβ) through activation of the NLRP3 inflammasome, a key innate immune sensor and a promising therapeutic target. *Leishmania infantum*, a protozoan parasite causing visceral leishmaniasis, is known to employ sophisticated mechanisms to subvert inflammatory responses in macrophages, including modulation of the NLRP3 inflammasome, thus representing a potential natural model for counteracting microglia-related inflammation. However, microglia-*Leishmania* interactions remain unexplored, particularly the parasite’s ability to modulate microglial NLRP3 activation. Here, we demonstrate that *L. infantum* invades and persists in microglia without inducing cell activation, indicating an immunologically silent entry. Aβ-stimulated NLRP3 activation was suppressed by *Leishmania* infection, as evidenced by a significant reduction in key pro-inflammatory mediators, including IL-1β, IL-18, TNF-α, and neurotoxic nitric oxide. Mechanistically, *L. infantum* disrupted NLRP3 priming by interfering with NF-κB signaling and upregulating the negative regulator A20. Additionally, *L. infantum* limited ASC speck formation, caspase-1 activation and ROS production while preserving lysosomal integrity. These findings reveal, for the first time, an unrecognized inhibitory effect of *L. infantum* on the microglial NLRP3/NF-κB axis and provide mechanistic insights into the parasite’s immune subversion in Aβ-activated microglia. Deciphering the molecular pathways exploited by *L. infantum* and the specific parasitic effectors involved could offer novel therapeutic targets and bioinspired strategies to mitigate microglial inflammatory responses in the context of AD.

**Supplementary Information:**

The online version contains supplementary material available at 10.1186/s12974-025-03574-5.

## Background

Innate immune activation and chronic neuroinflammation are hallmark pathological features of Alzheimer's disease (AD) [34], the most prevalent neurodegenerative disorder and the leading cause of dementia. Currently affecting approximately 50 million people globally, AD cases are expected to rise to 152 million by 2050 [60]. Despite its widespread prevalence, the etiology and pathogenesis of AD remain incompletely understood, resulting in inefficient treatments that primarily target symptoms rather than the underlying disease mechanisms. Among the cellular processes involved, microglial activation and inflammatory signaling are thought to contribute significantly to disease pathology. In particular, microglia, the brain’s resident macrophages, interact with amyloid-beta peptide (Aβ), promoting inflammatory cascades that contribute to the formation of neurofibrillary tangles and ultimately exacerbate neuronal dysfunction and cognitive decline [36].

Microglia play a dual role in AD pathology. While initially protecting the brain through Aβ phagocytosis and clearance, chronically activated microglia due to persistent Aβ deposition sustain inflammatory responses that accelerate disease progression [43, 72]. Intracellular Aβ activates signaling platforms known as inflammasomes, particularly the NLRP3 inflammasome, a cytosolic multiprotein complex that senses Aβ as a damage-associated molecular pattern (DAMP) [29]. This process involves a two-step mechanism: a priming phase, during which NF-κB (a central inflammatory transcription factor and key regulator of the inflammasome), upregulates the transcription of both *Nlrp3* and *Il1β* [47], followed by an activation phase leading to ASC oligomerization, caspase-1 activation, and the release of pro-inflammatory cytokines IL-1β and IL-18 [64].

The pathological role of microglial NLRP3 in perpetuating AD-related neuroinflammation has been validated in animal models, patient-derived cells [71] and post-mortem brain analyses [33], highlighting this pathway as a potential therapeutic target [73, 80]. NLRP3 activation is also involved in other neurodegenerative and neurodevelopmental conditions, including Parkinson’s disease, multiple sclerosis (MS), and autism spectrum disorder (ASD) [64]. Interestingly, the druggability of NLRP3 has been recently demonstrated in (neuro) inflammatory disorders such as Parkinson’s disease, amyotrophic lateral sclerosis (ALS) or cardiovascular disease [13]. However, effective therapeutic strategies that modulate NLRP3 activation in immune cells in AD remain elusive.

*Leishmania* parasites, obligate intracellular pathogens responsible for complex immunopathologies termed leishmaniasis [38], represent a unique biological model for immune modulation. Having co-evolved within macrophages, their primary cell niche, these eukaryotic parasites employ sophisticated strategies to manipulate macrophage cell signaling, immune functions, and metabolism to establish favorable conditions for long-term infections [62, 69, 75]. Notably, *Leishmania* exhibits potent anti-inflammatory properties, including the negative modulation of the NLRP3 inflammasome [18, 28, 40, 42, 74]. Among *Leishmania* species, visceralizing parasites such as *Leishmania infantum* and *L. donovani* are especially immunosuppressive, persisting in internal organs of the host like the spleen, liver and bone marrow (key components of the mononuclear phagocyte system), by subverting systemic immune responses [41]. Remarkably, these parasites are also capable of accessing the brain during visceral leishmaniasis infections, as shown in both experimental animal models and human cases [51, 54], However, whether visceral *Leishmania* species directly interact with microglia and modulate their inflammatory pathways remains unexplored.

Emerging evidence suggests an intriguing epidemiological link between reduced dementia prevalence and parasitic infections. Trumble et al. [78] identified a positive correlation between high parasite burdens and improved cognitive function in a Bolivian indigenous tribe, suggesting a possible protective role against AD progression [78]. This finding was further supported by epidemiological studies demonstrating exceptionally low dementia prevalence among the Tsimane and Moseten tribes, populations with high rates of parasitic infections [24]. This connection underscores the need to investigate *L. infantum* (endemic in Bolivia and many Latin American countries), as a natural biological system exerting potential neuroprotective properties.

Our previous work demonstrated that *L. infantum* inhibits NLRP3 activation in THP-1 cells exposed to Aβ [70]. Based on these findings, we hypothesized that the parasite’s anti-NLRP3 abilities might also be conserved in microglia. Using both primary microglia and the microglial cell line originally used to identify the role of NLRP3 in Aβ-induced immune responses [29], we investigated how *L. infantum* modulates inflammatory responses in Aβ-stimulated microglia. Our results revealed novel parasite-driven immune subversion mechanisms targeting the NLRP3/NF-κB axis, offering molecular insights for potential bioinspired modulation of microglial inflammatory responses relevant to AD.

## Results

### *L. infantum* infects microglia without inducing cell activation

**Fig. 1:**
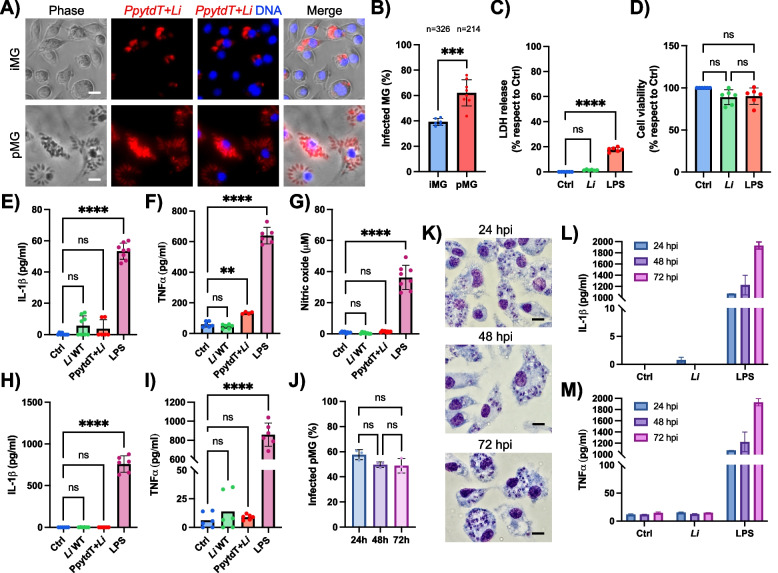
Immunologically silent entry of *L. infantum* intro microglia.** A** Representative fluorescence images of immortalized microglia (iMG) and primary microglia (pMG) infected with *L. infantum* parasites expressing the red-shifted luciferase PpyRE9H and the red fluorescent protein tdTomato (*PpytdT* + *Li*) after 24 hpi. DAPI staining of DNA (blue). Scale bars represent 10 μm. **B** Quantification of the proportion of infected MG after 24 hpi with *PpytdT* + *Li* parasites. The number of cells considered for quantification (n) is indicated above the graph. **C, D** Assessment of *Li*-induced cytotoxicity through the LDH assay (**C**) and the microglial cell viability by the MTT assay (**D**) of pMG. **E, F, G** Detection by ELISA of secreted IL-1β (**E**) and TNF-α (**F**), and released nitric oxide (**G**) by the Griess assay in immortalized microglia supernatants following infection with *Li* WT and transgenic *PpytdT* + *Li* after 24 h. Treatment with LPS (100 ng/ml) was used as a positive control for microglia activation (mean ± SD, *n* = 4–8; **p < 0.005, ****p < 0.0001). **H, I** Detection of IL-1β (**H**) and TNF-α (**I**) cytokines in primary microglia infected with *L. infantum* and stimulated with LPS (mean ± SD, *n* = 4; ****p < 0.0001). **J** Proportion of primary microglia infected with *Li* WT parasites after 24, 48 and 72 h. **K** Selected images stained with Giemsa from J. Scale bars: 10 μm. **L, M** IL-1β (**L**) and TNF-α levels (**M**) in the supernatants of primary microglia after 24, 48 and 72 h of infection with *Li* WT parasites. As a positive control for cytokine release, cells were treated with LPS (100 ng/ml) for the same periods of time in the absence of the parasites. Data were collected from three (**B**, **H**, **I**, **J**, **L** and **M**) or four (**C**-**G**) independent experiments (*n* = 3–4). All graphs are presented as mean ± SD and were analyzed by two-tailed, unpaired t test (**B**) and one-way ANOVA (**C**–**J**, **L**-**M**) in conjunction with Tukey’s multiple comparisons test (*p < 0.05; **p < 0.01; ***p < 0.001; ****p < 0.0001; ns, not significant).

We first evaluated the fate of *L. infantum* parasites in microglia and their ability to trigger an inflammatory response. Murine primary microglia (pMG) isolated from postnatal C57BL/6 WT mouse brains, and the immortalized mouse microglial cell line (iMG) originally employed to characterize NLRP3 inflammasome activation in response to Aβ [29], were used as microglial models. Notably, iMG expressed classical microglial surface markers of pMG (Fig. S1A-B). Microglia were infected with stationary-phase promastigotes of *L. infantum*, which include infectious metacyclic forms (the stage transmitted by the insect vector and efficiently phagocytosed by host macrophages). Two strains of *L. infantum* (*Li*) were used: a WT strain and a transgenic line stably expressing the bioluminescent marker PpyRE9H fused to the fluorescent reporter tdTomato (*PpytdT* + *Li*) [10]. Our results show that both iMG and pMG efficiently phagocytosed *PpytdT* + *Li* parasites after 24 hours post infection (hpi), as indicated by the presence of rounded, red fluorescent intracellular amastigotes (the replicative form within host macrophages), inside microglial cells (Fig. 1A). The proportion of infected microglia differed significantly, with pMG showing a higher infection rate (60%) compared to iMG (39%) (Fig. 1B), consistent with the enhanced capacity of primary cells to support intracellular parasite survival and proliferation [30]. The infection did not affect pMG viability, as evidenced by the absence of changes in LDH release and metabolic activity after 24 hpi (Fig. 1C-D), contrasting with the cytotoxicity observed in cells stimulated with bacterial lipopolysaccharide (LPS).

We next examined whether *L. infantum* infection stimulated the production of pro-inflammatory IL-1β and TNF-α cytokines, as well as that of nitric oxide (NO), key mediators of microglial inflammatory and neurotoxic responses [14, 77, 83]. The parasites did not induce the production of IL-1β (Fig. 1E, H) and TNF-α (Fig. 1F, I) in either microglial cell types, or the release of NO by iMG (Fig. 1G) at 24 hpi. Furthermore, infection of iMG resulted in a reduction in the expression of ionized calcium-binding adaptor molecule 1 (Iba-1), a marker of microglial activation (Fig. S1C-E). Given the parasite’s chronic immunosuppressive potential, pMG analyses were extended to 48 and 72 hpi. In line with earlier time points, results confirmed that parasites do not activate microglia over time (Fig. 1L-M). Additionally, the proportion of pMG harboring intracellular parasites remained stable across 24, 48 and 72 hpi, indicating sustained intracellular persistence (Fig. 1J-K).

In summary, these findings indicate that microglia efficiently phagocytose *L. infantum* and promote parasite survival while remaining in a non-activated state. This suggests an immunologically silent entry mechanism that allows the parasite to evade microglial inflammatory responses and activation.

### *L. infantum* suppresses the release of NLRP3-related mediators in Aβ-stimulated microglia in a parasite-dependent manner

We next investigated whether Aβ-induced NLRP3 activation of microglia and the associated inflammatory response might be inhibited by *L. infantum* infection. To this end, microglia were primed with LPS and stimulated with Aβ [29] in the presence or absence of the parasites. Nigericin was used as a canonical NLRP3 activator following LPS priming [56].

**Fig. 2:**
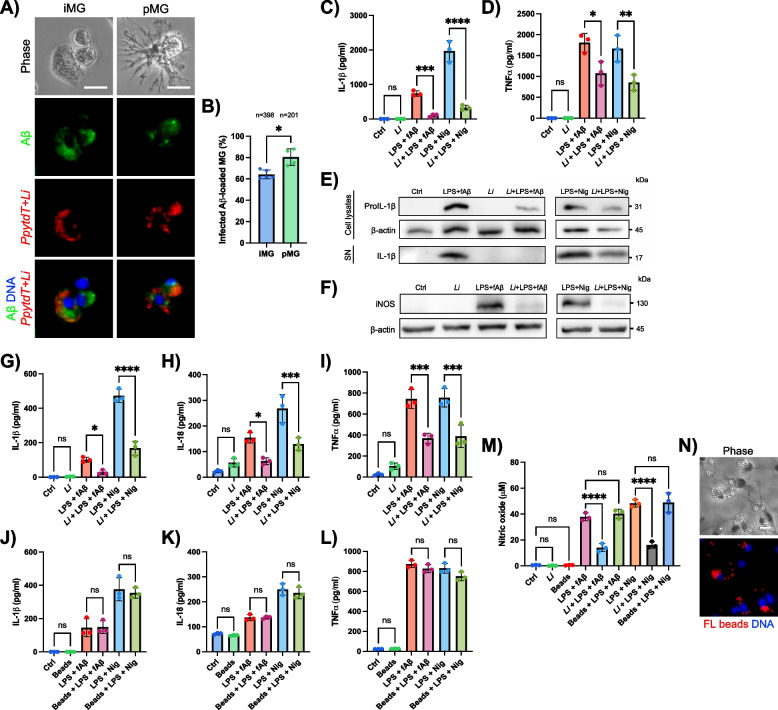
Prevention of the release of NLRP3-related inflammatory mediators by *L. infantum* in infected, Aβ-stimulated microglia.** A** Live-cell fluorescence images of immortalized (iMG) and primary microglia (pMG) loaded with a fluorescently-labeled Aβ peptide (green), and infected with *PpytdT* + *Li* parasites (red); DAPI staining (blue) to visualize the DNA content. Scale bars represent 10 µm. **B** Proportion of iMG and pMG positive for the presence of both green fluorescent Aβ and red fluorescent parasites. The number of cells used for quantification (n) is indicated above the graph. **C**,** D** Detection by ELISA of secreted IL-1β (**C**) and TNF-α (**D**) cytokines in the supernatants of infected pMG following LPS/Aβ or LPS/Nig stimulation. **E**,** F** Western blot analysis of pro-IL-1β and iNOS in primary microglial lysates, and mature IL-1β in culture supernatants (SN) from pMG. **G**,** H**,** I** Levels of secreted IL-1β (**G**), IL-18 (**H**) and TNF-α (**I**) quantified by ELISA in supernatants of iMG stimulated with LPS/Aβ or LPS/Nig. **J**, **K**,** L** Levels of secreted IL-1β (**J**), IL-18 (**K**) and TNF-α (**L**) quantified by ELISA in supernatants of iMG loaded with inert latex beads and stimulated with LPS/Aβ or LPS/Nig. **M** NO levels quantified by the Griess assay in iMG in the presence of *L. infantum* or inert latex beads and stimulated with LPS/Aβ or LPS/Nig. **N** Live-cell fluorescence images of iMG showing phagocytosed fluorescent inert latex beads (red), and DAPI staining for DNA content. Scale bar is 10 µm. Results represent the mean ± SD of three independent experiments (*n* = 3) with two technical replicates per assay (*n* = 6). Statistical tests included two-tailed, unpaired t test (**B**), and one-way ANOVA with Tukey’s ad-hoc post-tests for multiple comparisons (**C**-**D**, **G**-**I**, **J**-**M**). Levels of significance are indicated as follows: (***p < 0.005; ****p < 0.0001; ns, not significant).

Initial results obtained using a fluorescently-labeled Aβ peptide confirmed that both iMG and pMG effectively phagocytosed and harbored Aβ and *L. infantum* parasites within the same cell (Fig. 2A). The quantification of the infection rates in the presence of Aβ revealed a significantly higher infection index in pMG compared to the microglial cell line (Fig. 2B), consistent with prior observations (Fig. 1B). Notably, Aβ exposure enhanced the infection rate in both microglial cell models, suggesting that Aβ increases parasite uptake, potentially reflecting an increased microglial phagocytic activity.

We then assessed whether *L. infantum* infection suppresses the production of IL-1β, IL-18, TNF-α and NO upon NLRP3 activation. Infected LPS/Aβ- and LPS/Nig-stimulated pMG exhibited a significant reduced secretion of IL-1β and TNF-α (Fig. 2C-D), while IL-18 and NO levels remained undetectable (not shown). Additionally, pMG lysates showed a marked reduction in pro-IL-1β expression in cell cytoplasm of infected cells, along with a decrease in cleaved IL-1β levels in pMG supernatants (Fig. 2E). Consistent with this anti-inflammatory effect, the presence of the parasites was also associated with a downregulation of inducible nitric oxide synthase (iNOS) expression following NLRP3 stimulation (Fig. 2F). This effect was conserved in iMG, as we observed reduced IL-1β, IL-18, TNF-α and NO levels upon infection (Fig. 2G, H, I, M). Importantly, this resulted to be parasite-specific as increasing microglia-to-*L. infantum* ratios were inversely correlated with the levels of IL-1β, IL-18, TNF-α and NO (Fig. S2A-D).

To test whether phagocytosis alone could mediate this effect, iMG were exposed to inert fluorescent latex beads under LPS/Aβ or LPS/Nig stimulation. Unlike *L. infantum*-infected cells, bead uptake failed to reduce the pro-inflammatory response, as IL-1β, IL-18, TNF-α, and NO levels remained elevated and unchanged (Fig. 2J-N). This suggests that the observed anti-inflammatory effect is parasite-specific rather than a general consequence of particle internalization.

To further confirm that parasite internalization is required for this anti-inflammatory phenotype, iMG were treated with the phagocytosis inhibitor cytochalasin D (CytoD), prior to Aβ stimulation and parasite exposure. Confirming previous reports [29], CytoD significantly reduced Aβ-induced IL-1β production, including the downregulation of IL-1β transcript levels (Fig. S3A and B). As a positive control for IL-1β secretion, Aβ-stimulated iMG were treated with the caspase-1-specific inhibitor Ac-YVAD-cmk, resulting in the inhibition of IL-1β release. Remarkably, when CytoD was added after Aβ stimulation but before *L. infantum* infection, phagocytosis of *L. infantum* was nearly abolished (Fig. S3C), resulting in the loss of the parasite’s immunosuppressive effect and in a significant increase in IL-1β mRNA and protein levels (Fig. S3A and B). These results support the concept that suppression of inflammation is ensued by *L. infantum* internalization in microglial cells.

### *L. infantum* downregulates NF-κB signaling and NLRP3 priming by engaging the negative regulator A20

**Fig. 3:**
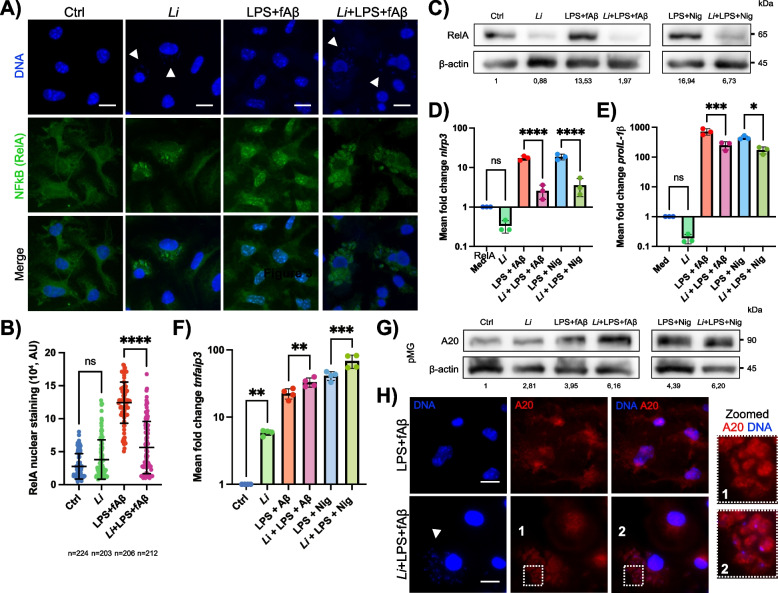
*L. infantum* prevents NLRP3 inflammasome priming in microglia by engaging the negative regulator A20. **A** Normalized immunofluorescence images of PFA-fixed primary microglia (pMG) stimulated or not with LPS/Aβ in the presence or the absence of the parasites and stained with an antibody anti-NF-κB p65 (RelA) (green). DNA was stained with DAPI (blue). White arrowheads show the parasite’s DNA within microglia. Scale bars represent 10 µm. **B** Quantitative evaluation of nuclear translocation of RelA by measuring the green fluorescence intensity in the nuclei of microglial cells after 24 h of stimulation and/or infection. The number of cells used for quantification (n) is indicated under the graph. **C** Relative quantitation by WB analysis of NF-κB RelA in total protein extracts of uninfected and infected pMG stimulated with LPS/Aβ and LPS/Nig for 24 h. Beta-actin was used as a control protein. **D, E, F** Transcriptional modulation of *nlrp3*, *proIL-1β* and *tnfaip3* in pMG after 24 h of infection and/or stimulation by qRT-PCR. **G** Western blot analysis and relative quantification of TNFAIP3/A20 in cell lysates of pMG. **H** Representative and normalized immunofluorescence pictures of pMG labelled with an anti-A20 antibody (red). DAPI staining for DNA content (blue). White arrowheads indicate the genetic material of the parasites. White boxes (zoomed panels 1 and 2) show the magnification of phagolysosomes containing intracellular amastigotes co-localizing with A20 and DAPI fluorescent signals. Scale bars show 10 µm. Results represent the mean ± SD of three independent experiments (*n* = 3) with two technical replicates per assay (*n* = 6). Statistical differences according to one-way ANOVA and Tukey’s comparison tests (*p < 0.05; **p < 0.01; ***p < 0.001; ****p < 0.0001; ns, not significant).

To investigate how *L. infantum* dampens NLRP3-related inflammatory responses in microglia, we examined the effect of infection on NF-κB p65 (RelA), a central transcription factor crucial for the priming phase of NLRP3 activation [47]. *Leishmania* parasites are known to disrupt NF-κB signaling to promote survival and persistence in macrophages [25, 42]. In pMG, LPS/Aβ stimulation induced a massive nuclear translocation of RelA (Fig. 3A, B), whereas this process was significantly reduced in Aβ-stimulated and infected cells (Fig. 3A, B). Notably, the presence of the parasites alone did not induce nuclear translocation of the transcription factor, showing a pattern similar to unstimulated control cells (Fig. 3A, B), a finding that was also confirmed in iMG (Fig. S4A). Furthermore, *L. infantum* infection significantly reduced total RelA protein expression in LPS/Aβ- and LPS/Nig-stimulated pMG (Fig. 3C), with a similar inhibition in the microglial cell line (Fig. S4B), suggesting that parasites actively downregulate RelA levels in response to pro-inflammatory stimuli. Compatible with the transcriptional activation of *Nnlrp3* and *Il1β* genes by NF-κB [47], we next examined whether *L. infantum* modulates their expression. Indeed, the transcription of both genes was significantly reduced in infected pMG and iMG following stimulation (Fig. 3D-E and Fig. S4C-D, respectively), indicating that *L. infantum* impairs inflammasome priming.

One mechanism by which *Leishmania* inhibits NF-κB signaling and NLRP3 activation in macrophages is by upregulating A20 (also known as TNF-α-induced protein 3, TNFAIP3), a host deubiquitinating enzyme that negatively regulates NF-κB [28, 42]. In line with this, we observed a significant increase in *Tnfaip3* transcript levels in *L. infantum*-infected pMG and a greater upregulation in infected microglia stimulated with LPS/Aβ or LPS/Nig (Fig. 3F). At the protein level, we observed a twofold increase in total A20 protein expression in infected and stimulated pMG (Fig. 3G), demonstrating that *L. infantum* alone is sufficient to drive A20 upregulation.

A20-dependent termination of NF-κB signaling has been linked to its localization within lysosome-associated compartments [44], where it targets signaling molecules for degradation [45]. Since *Leishmania* amastigotes reside within parasitophorous vacuoles (PVs) formed through the fusion of phagosomes with lysosomes [8], we next analyzed the subcellular localization of A20 in infected cells. Immunofluorescence analyses showed that A20 localizes to *L. infantum*-containing phagolysosomes in pMG, as indicated by rounded structures co-localizing with intracellular amastigotes (Fig. 3H). These findings suggest that *L. infantum* may exploit the degradative functions of A20 within lysosomal compartments to terminate NF-κB signaling and impair NLRP3 priming in microglia, revealing a novel immune subversion strategy employed by the parasite.

### *L. infantum* interferes with ASC and caspase-1 to prevent NLRP3 inflammasome activation

Following the priming step, a second signal leads to assembly and activation of the inflammasome complex. This process requires the recruitment of the adaptor protein ASC and the effector caspase-1, leading to its autoproteolytic activation. During this phase, ASC assembles into large perinuclear fibrillar specks [49], which can be found intracellularly or secreted into the extracellular space. Notably, ASC specks released by activated microglia can bind to Aβ, enhancing its cerebral aggregation and deposition as well as the spreading of Aβ brain pathology [81].

**Fig. 4:**
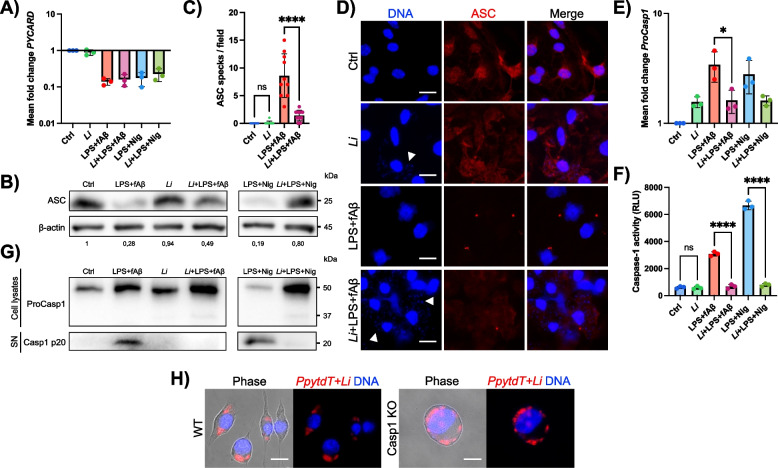
The presence of *L. infantum* inhibits the formation of ASC specks and the activity of caspase-1 in Aβ-stimulated microglia.** A** Transcript modulation of *PYCARD* in primary microglia (pMG) as assessed by RT-qPCR. **B** Western blot analysis and relative quantification of ASC in pMG lysates from control, LPS/Aβ- and LPS/Nig-stimulated samples in the presence or absence of the parasites. Beta-actin was used as a control protein. **C, D** Quantification of the number of ASC specks (**C**) in immunofluorescence images of pMG stained with an anti-ASC antibody (red) to reveal the presence of ASC specks after 24 h of infection and/or stimulation with LPS/Aβ (**D**). DNA content in blue; white arrowheads show the parasite’s DNA. Scale bars show 10 µm. **E** Transcriptional modulation of *ProCasp1* in pMG. **F** Enzymatic caspase-1 activity by the Caspase-Glo 1 Inflammasome assay in uninfected and infected pMG treated or not with LPS/Aβ and LPS/Nig. **G** Western blot analysis of ProCasp1 and cleaved Casp1 p20 in pMG lysates and culture supernatants (SN), respectively. **H** Representative live-cell fluorescence images of immortalized WT microglia and caspase-1-deficient microglia infected with red fluorescent *PpytdT +Li* parasites. DAPI staining of DNA content (blue). Scale bars show 10 µm. Data were collected from three independent experiments (*n* = 3) with duplicate (**A**, **C**, **E**, **H**; *n* = 6) or triplicate (**F**; *n* = 9) technical replicates for all conditions. Graphs are presented as mean ± SD and were analyzed by one-way ANOVA in conjunction with Tukey’s test (****p < 0.0001; ns, not significant).

We next investigated whether *L. infantum* infection could interfere with core NLRP3 molecular components, including ASC and caspase-1. At the mRNA level, no significant changes were observed in the transcript abundance of *PYCARD* (the gene encoding ASC) in *L. infantum*-infected pMG stimulated with LPS/Aβ or LPS/Nig (Fig. 4A). In contrast, ASC protein expression was reduced in LPS/Aβ- and LPS/Nig-treated pMG, but increased in the presence of *L. infantum*, suggesting a lack or reduced ASC oligomerization mediated by the parasites (Fig. 4B) [20]. Of note, ASC protein levels remained unchanged in both control and infected cells. Based on these results, we further assessed ASC speck formation in activated pMG by immunofluorescence. Data showed that *L. infantum* infection significantly reduced the number of ASC puncta in LPS/Aβ-stimulated pMG, indicating that the parasites impair ASC oligomerization and speck formation (Fig. 4C, D). Remarkably, *L. infantum* alone did not induce ASC speck formation, as evidenced by its diffuse cytoplasmic localization and the absence of specks in infected cells (Fig. 4C, D). These findings were further validated in iMG by imaging flow cytometry, confirming the reduction of intracellular ASC specks in infected cells (Fig. S5).

In parallel, *L. infantum* infection slightly increased *ProCasp-1* transcript levels in unstimulated pMG, while reducing its expression in LPS/Aβ- or LPS/Nig-stimulated cells (Fig. 4E). These findings, combined with the observed inhibition of IL-1β and IL-18 secretion in infected microglia upon LPS/Aβ or LPS/Nig stimulation (Fig. 2), suggest three potential mechanisms: 1) impaired caspase-1 autoproteolysis, 2) reduced expression of activated caspase-1, and/or 3) the inhibition of caspase-1-mediated cleavage of pro-IL-1β and pro-IL-18. Supporting hypotheses 2 and 3, caspase-1 enzymatic activity was significantly decreased in infected pMG stimulated with LPS/Aβ or LPS/Nig (Fig. 4F). Additionally, the infection also resulted in the absence of active caspase-1 p20 in LPS/Aβ-stimulated pMG, and a marked reduction in caspase-1 activation in LPS/Nig-stimulated cells (Fig. 4G). Finally, by infecting caspase-1-deficient (KO) microglia vs the WT cell line, a higher number of intracellular amastigotes was observed in caspase-1 KO cells, indicating that the absence of caspase-1 facilitates parasite proliferation (Fig. 4H). Collectively, these data show that *L. infantum* prevents ASC speck formation and inhibits caspase-1 activation, thereby dampening NLRP3 activation in Aβ-stimulated microglia.

### *L. infantum* reduces ROS generation and preserves lysosomal integrity to prevent NLRP3 activation

**Fig. 5:**
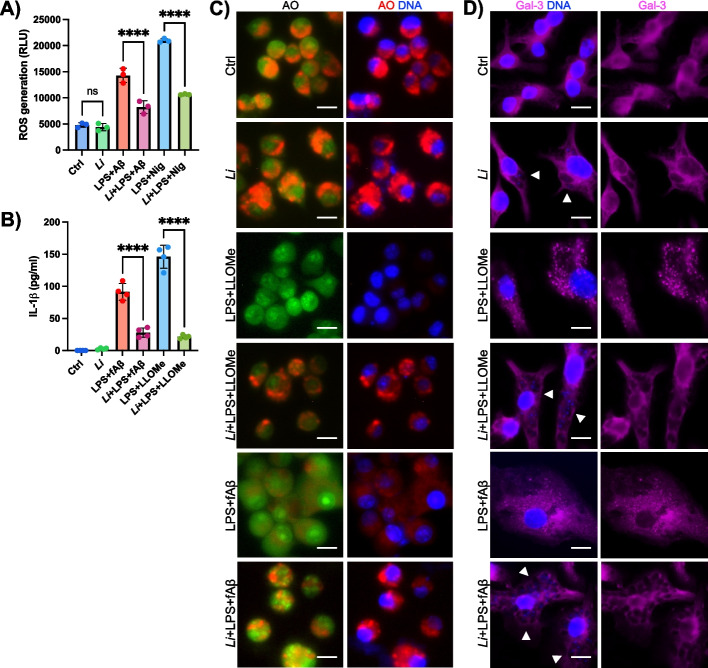
The activation of the microglial NLRP3 by mitochondrial and lysosomal damages is prevented by *L. infantum* parasites.** A** Production of ROS by immortalized microglia (iMG) as assessed by the ROS-Glo assay kit under NLRP3 activating conditions in infected and uninfected cells. **B** ELISA of the release of IL-1β by Aβ-stimulated iMG in the presence or the absence of the parasites, and upon treatment with the LMP inducer LLOMe. **C** Normalized live-cell fluorescence images showing the lysosomal permeabilization assay in iMG through the staining with acridine orange (AO). Red fluorescence indicates acidic compartments (intact lysosomes), whereas the loss of red signal and increase in green fluorescence show leakage of AO into the cytosol and LMP. DAPI staining of DNA content (blue). Scale bars represent 10 µm. **D** Selected immunofluorescence images of iMG stained with an anti-Gal-3 antibody (magenta) after infection with *L. infantum* parasites and stimulation with LPS/LLOMe and LPS/Aβ. DAPI staining of DNA content (blue). White arrowheads showing the presence of *L. infantum* within intact phagolysosomes (empty rounded structures) and the parasites’ DNA. Scale bars: 10 µm. Results are representative of independent experiments performed three times (*n* = 3) with two technical replicates (*n* = 6). Graphs are presented as mean ± SD and were analyzed by one-way ANOVA in conjunction with Tukey’s test (****p < 0.0001; ns, not significant).

Aβ species induce mitochondrial and lysosomal damage in microglia leading to the release of mitochondrial reactive oxygen species (ROS) and lysosomal contents [12, 29]. These events serve as the second signal for NLRP3 inflammasome activation [84]. To gain deeper insights into the molecular mechanisms by which *L. infantum* inhibits NLRP3 activation, we assessed its impact on ROS production and lysosomal rupture in microglia. *Leishmania* parasites are known to suppress NLRP3 activation in macrophages by inhibiting ROS generation [74]. Consistent with this, we observed a significant reduction in ROS levels in *L. infantum*-infected iMG stimulated with LPS/Aβ or LPS/Nig (Fig. 5A). Next, we verified whether *L. infantum* could counteract Aβ-induced lysosomal damage and prevent NLRP3-mediated inflammation by suppressing lysosomal membrane permeabilization (LMP), a process which results in the release of lysosomal contents from damaged lysosomes into the cytosol, further promoting inflammation. To this end, we stimulated iMG with LPS/Aβ or LPS/L-Leucyl-L-Leucine methyl ester (LLOMe), a potent LMP inducer that serves as the second signal for NLRP3 activation and IL-1β release [16]. We found that *L. infantum* infection significantly inhibited IL-1β secretion in microglia treated with LPS/Aβ or LPS/LLOMe (Fig. 5B), suggesting that the parasite protects against LMP induction and LMP-dependent inflammatory signaling.

To directly assess LMP, we used the weak lysosomotropic dye acridine orange (AO), which accumulates in intact lysosomes emitting red fluorescence and shifts to green upon lysosomal membrane rupture [3]. *L. infantum*-infected iMG exhibited fluorescence patterns comparable to that of control cells (Fig. 5C), indicating the presence of intact lysosomes. In contrast, LPS/LLOMe treatment markedly reduced red fluorescence resulting in a predominant green signal, consistent with LMP induction. Remarkably, *L. infantum* infection restored the red fluorescent signal in LLOMe-treated cells (Fig. 5C). Similarly, *L. infantum* rescued the red fluorescent signal in LPS/Aβ-treated microglia, indicating a protective effect against LMP (Fig. 5C). To further validate these results, we analyzed the subcellular localization of galectin-3 (Gal-3), a highly sensitive marker to detect lysosomal membrane damage and monitor LMP [1, 3]. As expected, rapid Gal-3 recruitment to damaged lysosomes was observed following LPS/LLOMe treatment, and to a lesser extent in LPS/Aβ-stimulated iMG (Fig. 5D), as evidenced by the presence of Gal-3 puncta. Interestingly, *L. infantum* infection completely prevented Gal-3 recruitment to damaged lysosomes, resulting in Gal-3 excluded from parasite-containing and structurally intact phagolysosomes, even in LPS/LLOMe- and LPS/Aβ-treated cells (Fig. 5D).

Altogether, these results demonstrate that *L. infantum* reduces cellular oxidative stress and preserves lysosomal integrity in microglia, thereby preventing key cellular events required for NLRP3 activation. This adds an additional layer of complexity to the strategies used by *L. infantum* to evade innate immune pathways, expanding its molecular repertoire to dampen NLRP3 activation and inflammation in Aβ-activated microglia.

## Discussion

This study uncovers a novel and previously unrecognized role of *Leishmania infantum* in modulating NLRP3 inflammasome activation and associated inflammatory signaling pathways in Aβ-stimulated microglia. By downregulating key molecular components of the NLRP3/NF-κB axis, *L. infantum* subverts the inflammatory responses of microglia, offering mechanistic insights that may inform the development of future biotherapeutic strategies. The ability of *L. infantum* to persist in microglia without triggering activation highlights a parasite’s evolutionary conserved strategy of immunologically silent entry, previously described in peripheral macrophages [11, 38, 62, 75], and now extended to brain-resident innate immune cells. This observation is particularly relevant given the unique developmental origin and transcriptional and functional identities of microglia compared to monocyte-derived macrophages [2, 15, 26]. Our findings provide the first direct evidence of *L. infantum*’s interactions with microglia, expanding the parasites’ immunomodulatory repertoire to include brain macrophages.

For the first time, our data demonstrate that *L. infantum* infection reduces the inflammatory phenotype of Aβ-activated microglia, as evidenced by the reduction in NLRP3-related cytokines and nitric oxide, which are pivotal inflammatory mediators implicated in Aβ-induced cytotoxicity and neuroinflammation [14, 80, 83]. While we used insect-stage promastigotes for microglial infection, prior studies have shown that amastigote forms induce weaker inflammasome activation in macrophages [17, 19, 42]. It remains to be investigated whether the intracellular stage of *L. infantum* exerts an even stronger anti-inflammatory effect in microglia.

Interestingly, in contrast to our findings showing persistent infection and active immunosuppression of microglia by *L. infantum*, Ramos et al. [68] reported progressive clearance of *L. braziliensis* and *L. amazonensis* from primary microglial cultures, accompanied by distinct ultrastructural alterations and cytokine profiles [68]. These discrepancies may reflect species-specific differences in tissue tropism and immune evasion strategies, as *L. braziliensis* and *L. amazonensis* are typically associated with cutaneous leishmaniasis. Variations in host genetic background may have also contributed to the divergent outcomes. Altogether, these observations underscore the influence of both parasite species and host factors in shaping microglial infection dynamics and the resulting inflammatory responses.

The observed downregulation of IL-1β, IL-18 and TNF-α, coupled with inhibited NF-κB activation, underscores *L. infantum*’s ability to suppress key microglial inflammatory drivers [9, 31, 31, 32, 32, 37, 43]. Notably, A20 (TNFAIP3), a central negative regulator of NF-κB signaling [66], was upregulated by *L. infantum* infection both in unstimulated and Aβ-activated microglia, providing a mechanistic explanation for the inhibition of NLRP3 priming and potentially contributing to parasite survival. Indeed, this is consistent with prior reports showing a global inhibition of NF-κB-mediated pro-inflammatory responses and a transcriptional increase of A20 in *L. amazonensis*- [42] and *L. donovani*-infected macrophages [28] in the context of NLRP3 activation. Strikingly, we observed A20 co-localizing within *L. infantum*-containing phagolysosomes, suggesting that the parasite may exploit A20 to neutralize NF-κB activity and interfere with NLRP3 priming. This mechanism aligns with the localization of A20 within lysosome-associated compartments, where A20 terminates pro-inflammatory signaling by targeting key mediators such as TRAF2 for lysosomal degradation [44, 45]. In neurodegenerative disease models, A20 has been shown to prevent microglial activation and NLRP3-driven inflammatory pathways [82], further reinforcing its potential relevance in the context of AD-associated immune responses. Although the precise parasite effectors involved remain unknown, our findings support the hypothesis that *L. infantum* modulates microglial NLRP3 activation by leveraging molecules like A20 to attenuate the deleterious inflammatory cascade triggered by Aβ.

We also found that *L. infantum* infection disrupts NLRP3 assembly and activation, as evidenced by the reduction in ASC speck formation and caspase-1 cleavage, leading to decreased secretion of mature IL-1β and IL-18. These results highlight the parasite’s capacity to inhibit essential inflammasome processes, as previously observed in infected bone-marrow derived macrophages [19, 42]. This is particularly significant considering that ASC specks regulate caspase-1 activity [22] and, when released by NLRP3-activated microglia, form ASC-Aβ composites that amplify microglial toxicity and promote Aβ cross-seeding in AD [23, 81]. Thus, inhibition of ASC speck formation by *L. infantum* may represent an important mechanism to limit detrimental inflammatory loops in Aβ-challenged microglia.

A particularly intriguing observation is the restoration of lysosomal integrity in microglia infected with *L. infantum*. Indeed, the infection prevented the lysosomal damage induced by Aβ or LLOMe and counteracted NLRP3 activation by reducing oxidative stress and preserving lysosomal membrane integrity. While *Leishmania*-mediated inhibition of ROS production in macrophages is well-documented [5, 62, 63, 74], to our knowledge, its ability to restore lysosomal integrity represents a novel parasite-mediated mechanism. Lysosomes are crucial cytoplasmic acidic organelles involved in cellular degradation, phagocytosis and nutrient sensing [3, 50]. Lysosomal membrane permeabilization (LMP) results in the release of luminal hydrolases into the cytosol, leading to neurodegenerative processes [7, 79]. Moreover, Aβ accumulation disrupts lysosomal acidification in microglia, impairing their degradative capacity and perpetuating brain inflammation. [67]. In this context, the ability of *L. infantum* infection to preserve lysosomal integrity in Aβ-stimulated microglia could be key to limiting inflammatory signaling. This effect might be also linked to the parasite’s adaptation to reside in parasitophorous vacuoles, which originate from lysosomal fusion and provide a permissive niche for intracellular survival [8]. Additionally, the reduction in IL-1β levels in infected microglia treated with Aβ or LLOMe may reflect a decreased cytosolic leakage of cathepsin B, a lysosomal protease linked to inflammation and neurodegeneration [29, 35, 58, 59].

Although our findings offer new perspectives on *L. infantum*’s anti-inflammatory abilities in microglia, several limitations must be acknowledged. Most importantly, this work was conducted in vitro using isolated microglia, which do not fully recapitulate the complexity of the CNS microenvironment. Crosstalk among microglia, neurons and astrocytes is essential to understand how inflammatory signals evolve in neurodegenerative settings including in AD [6, 43]. In particular, microglia-astrocyte interactions modulate cerebral inflammatory responses [4], and their dysregulation exacerbates AD progression [46, 53]. Additionally, while neurons lack a pro-inflammatory machinery, microglial inflammation can induce widespread neuronal cell death, further worsening AD pathology [31, 32, 52]. Future studies should explore how *L. infantum*-infected microglia interact with other CNS cell types to estimate the impact of the parasites immunomodulatory mechanisms in more physiologically relevant models.

In parallel, transcriptomic and proteomic analyses will be essential to identify the full repertoire of microglial molecular pathways targeted by *L. infantum* and to provide a comprehensive map of *L. infantum*’s immunomodulatory effector molecules. This could guide the design of novel bioinspired therapeutics, such as synthetic NLRP3 inhibitors or new classes of anti-inflammatory compounds of parasite origin. Notably, the exploitation of parasite-derived immunomodulatory molecules to tackle immune-related disorders has recently gained attention [57]. Among these, *Leishmania* virulence factors such as GP63, a metalloprotease capable of cleaving host proteins including NLRP3 and NF‑κB [27, 61, 74], and lipophosphoglycan (LPG), a surface glycoconjugate that modulates immune signaling and impairs phagosome maturation, may serve as promising candidates to inhibit inflammasome activation [21, 48]. Importantly, recent clinical trials have confirmed the druggability of the NLRP3 in chronic inflammatory diseases [13]. Integrating parasite-based strategies into this framework may offer innovative tools to selectively modulate innate immune activation in neurodegenerative conditions. Finally, evaluating the potential neuroprotective effects of *L. infantum* using animal models of AD is a critical step to validate the translational potential of our findings.

**Fig. 6:**
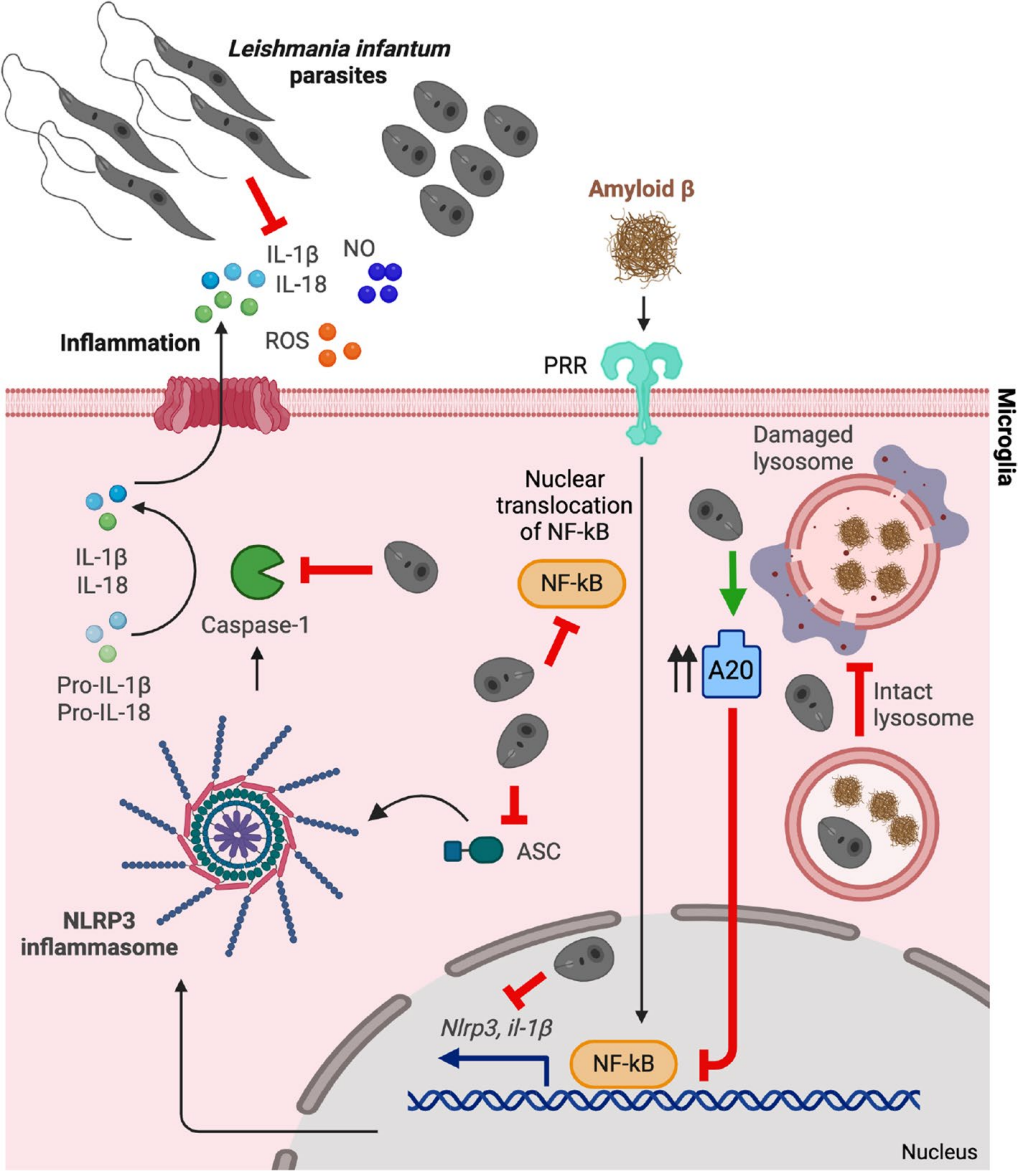
Immune subversion model of NLRP3 inflammasome suppression by *Leishmania infantum* in Aβ-stimulated microglia. *L. infantum* parasites enter microglia and persist as intracellular amastigotes without inducing cell activation, establishing an immunologically silent infection. In the presence of Aβ, infection markedly reduces the release of NLRP3-related cytokines and other inflammatory mediators by dampening NF-κB signaling during the priming step, partly through upregulation of the negative regulator A20. During the activation step, *L. infantum* prevents ASC speck formation and caspase-1 activation, thereby impairing inflammasome assembly and IL-1β/IL-18 maturation and release. Additionally, *L. infantum* reduces Aβ-induced mitochondrial ROS production and preserves lysosomal integrity, preventing lysosomal membrane permeabilization (LMP) and the release of lysosomal contents that act as NLRP3 activators. Collectively, these findings identify *L. infantum* as a natural model of NLRP3 inflammasome inhibition in microglia, revealing parasite-driven mechanisms of immune modulation that could inspire novel anti-inflammatory strategies in the context of AD.

In conclusion, this study defines new multifaceted mechanisms by which *L. infantum* subverts and modulates NLRP3 inflammasome signaling in Aβ-stimulated microglia (Fig. 6). This offers a conceptual basis for future investigations into bioinspired therapeutic development and parasite-derived immunomodulatory strategies in the context of AD.

## Materials and methods

### Reagents

Ultrapure LPS (*Escherichia coli* 0111:B4, tlrl-3pelps), nigericin (tlrl-nig), and Ac-YVAD-cmk (inh-yvad) were from InvivoGen. Amyloid β-protein (1–42) (HFIP-treated) was from Bachem (4090148), and HiLyte Fluor™ 488-labeled Aβ (1–42) was from AnaSpec (AS-60479). Acridine orange hemi(zinc chloride) salt (#158550), 4′,6-diamidino-2-phenylindole (DAPI, #D8417), Fluoroshield™ with DAPI (#F6057) were from Sigma Aldrich, and L-Leucyl-L-Leucine methyl ester hydrochloride (LLOMe) was from MedChemExpress (HY-129905). Antibodies against Iba-1 (clone E4O4W, #17198), NFκB p65 (clone D14E12, #BK8242S), iNOS (D6B6S, #13120), β-actin (clone 13E5, #4970), α-tubulin (#2144) and PE-ASC/TMS1 (D2W8U, #70891) were from Cell Signaling. FITC-NLRP3/NALP3 (768319, #FAB7578F) was from Novus Biologicals. ASC (AL177, AG-25B-0006) and Caspase-1 (Casper-1, AG-20B-0042) were from AdipoGen. CD68 (RM1031, ab303565) and TNFAIP3/A20 (EPR2663, ab92324) were from Abcam. Anti-IL-1β (B122, sc-12742) and Gal-3 (M3/38, sc-23938) were from Santa Cruz Biotechnology. Anti-F4/80 (clone BM8, 123102) was from BioLegend. Secondary antibodies HRP goat anti-Armenian hamster IgG (JI127035099), Cy3-conjugated goat anti-rabbit IgG (JI111-165-003), Alexa Fluor® 488-conjugated goat anti-mouse IgG, subclass 1 (JI115-545–205), and Cy3-conjugated goat anti-mouse IgG, subclass 2a (JI115-165-206) were from Jackson ImmunoResearch; Alexa Fluor® 555-conjugated goat anti-rat IgG (A-21434) was from Invitrogen, HRP goat anti-rabbit IgG (#7074) was from Cell Signaling, and mouse IgGκ light chain binding protein (m-IgGκBP, sc-516102) conjugated to HRP was purchased from Santa Cruz Biotechnology.

### Ethics statement

All animal studies were conducted according to the EU Directive 2010/63/EU and were reported in compliance with the ARRIVE 2.0 guidelines [39]. The experiments were designed based on the 3R principles of replacement, refinement and reduction. Animal care and experimental protocols were approved by the Italian Ministry of Research under number 5247B.N.UQP. Animals were housed in the animal care facility of the Department of Pharmacological and Biomolecular Sciences at the University of Milan, allowing them to food and water access ad libitum, and kept in temperature-controlled facilities on a 12-h light and dark cycle. C57BL/6 J mice (#000664) were supplied by Charles River Laboratories (Italy). Mouse breeding and husbandry were done according to the Italian animal welfare law.

### Microglia isolation and cell culture

For primary microglia isolation, the brains of 114 0–3-day-old C57BL/6 pups were used. Briefly, brains from neonatal mice (P0-P3, mixed gender), previously killed by decapitation without anesthesia, were stripped of the meninges and dissociated using mechanical shearing and trypsin digestion in a solution of 2.5% (Sigma-Aldrich) and 1% DNAse (Sigma-Aldrich), filtered through a 100-μm cell strainer, and seeded at the confluence of 30 × 10^6^ in a 75-cm^2^ flask in minimum essential Eagle’s medium (MEM) supplemented with 20% (v/v) heat-inactivated ultralow endotoxin fetal bovine serum (FBS, Euroclone), 0.6% glucose, 1% penicillin and streptomycin, and 1% L-glutamine (complete MEM). Glial cells were grown at 37 °C under a humidified 5% CO_2_ and 95% air atmosphere, and medium was replaced every 3 days. After 20 days, microglia were obtained by shaking the confluent monolayer of mixed glial cells at 260 rpm for 2 h and seeded in 24, 48 or 96-well plates at the confluence of 2.5 × 10^5^, 1 × 10^5^ or 0.5 × 10^5^ cells/well, respectively. The medium was changed with a mixture of 50% conditioning medium from the mixed glial culture and 50% of complete MEM 45 min after microglia plating to remove contaminating cells. The microglial cell lines (WT and caspase-1 knockout), were originally generated from primary cultures of C57BL/6 mouse microglia and subsequently immortalized using J2 recombinant retrovirus carrying the *v-myc* and *v-raf* oncogenes [29]. Cells were cultured in RPMI 1640 supplemented with L-glutamine, ciprofloxacin (Cellgro) and 10% (vol/vol) ultralow endotoxin FBS (Euroclone). *Mycoplasma* spp. contamination was excluded by regular testing.

### Parasite culture and generation of dual fluorescent-bioluminescent *L. infantum*

*Leishmania infantum* promastigotes (MHOM/TN/80/IPT1) (WHO international reference strain, kindly provided by Dr. M. Gramiccia and Dr. T. Di Muccio, ISS, Roma, Italy) were routinely cultured at 24 °C in Schneider’s insect medium (Sigma-Aldrich) supplemented with 2 mM L-glutamine and 10% (v/v) heat-inactivated, ultralow endotoxin fetal bovine serum (FBS, Euroclone) and antibiotic cocktail (200 U/mL penicillin, 200 μg/mL streptomycin). For generating the dual fluorescent-bioluminescent transgenic strain, parasites were cultured at 24˚C in M199 medium (Sigma), supplemented with 25 mM HEPES pH 6.9, 7.6 mM hemin, 10% (v/v) heat-inactivated FBS (Euroclone), and antibiotic mixture (200 U/mL penicillin and 200 μg/mL streptomycin). *L. infantum* parasites expressing the red-shifted luciferase (PpyRE9H) fused to the tdTomato red fluorescent protein by a Ty1 tag (*PpytdT* + *L. infantum*) were obtained after electroporation with pLEXSY-PpyRE9H-Ty1-tdTomato construct [10], and subsequent selection of transfectants in medium containing hygromycin (200 μg/mL, InvivoGen). Clonal populations were obtained by limiting dilution and cell culture growth was monitored with an automatic Muse cell analyzer (Merck Millipore).

### Fibrillar amyloid-β peptide preparation

Amyloid-β protein (1–42) was ordered 1,1,1,3,3,3-Hexafluoro-2-propanol (HFIP)-treated (Bachem AG, Switzerland) and stored at − 80 °C. Fibrillar Aβ was prepared according to the protocol of [76]. Briefly, HFIP-treated Aβ was dissolved in DMSO to a final concentration of 5 mM, thoroughly vortexed for 30 s, and sonicated for 10 min in a bath sonicator. This preparation is used as the starting material for unaggregated Aβ. Then, monomeric Aβ was added to 10 mM HCl to a final concentration of 100 μM, immediately vortexed for 15 s, and incubated for 24 h at 37 °C. As a working concentration 5 μM was used for cytotoxicity and cell viability assays, WB and ELISA. For phagocytosis and degradation assays, HiLyte Fluor™ 488-labeled Aβ (1–42) (AnaSpec, California, USA) was reconstituted in 1% NH_4_OH at 2 mg/ml, further diluted in PBS to 221 μM, incubated for 1 day at 37 °C and finally stored at − 80 °C.

### Microglia infection

*Leishmania infantum* WT or transgenic parasites expressing PpyRE9H-Ty1-tdT were grown in complete Schneider’s medium at high parasite densities and subcultured every 2–3 days. Parasites used for infections were let reach the stationary phase of growth (4–5 days of culture), added at a multiplicity of infection (MOI) of 5:1, 10:1, or 20:1 promastigotes:microglia, and incubated at 37 °C in a 5% CO_2_ atmosphere for up to 3 days post-infection. Microglia were also allowed to ingest fluorescent inert latex beads (5 μm particle size, Sigma-Aldrich) at a bead-to-microglia ratio of 20:1. Phagocytic activity was determined through Giemsa staining or using the red fluorescence emitted by living *PpytdT* + *L. infantum* parasites in infected, PFA-fixed microglia under a Nikon Eclipse Ti2 microscope.

### Cell viability and cytotoxicity assays

To assess the impact of *L. infantum* parasites on microglia viability and the parasite’s potential cytotoxicity to microglial cells, an MTT assay and an LDH cytotoxicity detection kit were used, respectively. As a positive control for microglia activation and cytotoxicity, cells were treated with 100 ng/ml LPS for 24 h. The MTT (3-[4,5-dimethylthiazol-2-yl]−2,5-diphenyltetrazolium bromide) assay was used with minor modifications [55]. After infection and/or treatment with LPS, cells were washed three times with PBS, followed by incubation in complete medium containing 5 mg/ml MTT solution for 3 h at 37 °C in a 5% CO_2_ atmosphere. The plates were then centrifuged, the supernatants discarded, and the formazan crystals dissolved in 100 μL of lysing buffer (20% w/v of a solution of SDS, and 40% of N,N-dimethylformamide in H_2_O). The following day, the absorbance was then measured spectrophotometrically at 550 nm (reference wavelength 650 nm) in a Synergy microplate reader (BioTek). To assess the cytotoxicity of the parasites to microglia, we used the LDH cytotoxicity assay kit (Cayman) according to the manufacturer's protocol. Absorbance was measured at 450 nm using the Synergy microplate reader (BioTek).

### NLRP3 inflammasome activation and lysosomal membrane permeabilization (LMP) in microglial cultures

Inflammasome activity was analyzed in microglia primed with 100 ng/ml LPS for 3 h and 5 μM fibrillar Aβ (1–42) for 21 h in medium containing 1% FBS. As a positive control, the inflammasome inducer nigericin was used at 10 μM concentration during the last 30 min or during the last 2 h for primary microglia and the microglial cell line, respectively. Ac-YVAD-cmk (1 μM), a specific capase-1 inhibitor, was added 1 h before infection and cell stimulation for NLRP3 activation. In some experiments, LMP was induced by exposing microglia to the lysosomotropic agent LLOMe (0.5 mM) for 30 min at 37 °C in a 5% CO_2_ atmosphere.

### Cytokine quantitation and nitric oxide measurement in culture supernatants

Cytokines were quantified in cell culture supernatants using mouse DuoSet ELISA kits for IL-1β, IL-18 and TNF-α (R&D Systems) according to the manufacturer’s instructions. Nitric oxide was measured by the Griess reaction, which quantifies nitrites (a stable end product of nitric oxide metabolism), accumulated in the cell supernatants with chemicals from Sigma.

### ROS and caspase-1 assays

The luminescent ROS-Glo assay kit (Promega) was used to measure hydrogen peroxide generation (H_2_O_2_) in stimulated and infected microglia according to the manufacturer instructions. Briefly, microglia were seeded at 5 × 10^4^ cells/well in white 96-well plates (Costar), let to adhere for 1 h at 37 °C in a 5% CO_2_ atmosphere, and immediately infected with *L. infantum* WT parasites at 10:1 parasites:cells ratio in MEM medium containing 0.5 mM sodium pyruvate and 1% FBS. After 16 h, non-phagocytosed parasites were extensively washed with PBS, followed by LPS priming (1 μg/ml) for 2 h in FBS-free medium. Subsequently, cells were then treated for 6 h with 5 μM fibrillar Aβ (1–42) together with the H_2_O_2_ substrate (25 µM). As a positive control, 10 µM nigericin was added the last two hours of the treatment period. After the incubation time, the ROS-Glo detection solution was added to the wells, incubated for 30 min at room temperature, and the relative luminescence units recorded using a Synergy HT plate reader (BioTek). On the other hand, caspase-1 activity was measured using the luminescent Caspase-Glo 1 Inflammasome Assay (Promega). It uses a luminogenic caspase-1 substrate (Z-WEHD-aminoluciferin), which upon caspase-1 cleavage releases aminoluciferin (luciferase substrate), resulting in the generation of light by a recombinant luciferase. Cell culture, infection and stimulation conditions were the same as described for the ROS-Glo assay. At the end of the treatment period, Caspase-Glo® 1 Reagent was added to the wells, incubated for 1 h at room temperature, and the relative luminescence units recorded using a Synergy HT (BioTek).

### Western blotting

Primary and immortalized microglia were lysed in RIPA buffer (89900, Thermo Scientific) containing protease/phosphatase inhibitors (78446, Thermo Scientific). Proteins were resolved by SDS–PAGE (10% or 15%), electroblotted in nitrocellulose membranes, blocked (5% fat-free milk, Tris-buffered saline, 0.25% Tween 20) and probed overnight at 4 °C with anti-iNOS (D6B6S, Cell Signaling), TNFAIP3/A20 (EPR2663, Abcam), NFκB p65 (D14E12, Cell Signaling), Iba-1 (E4O4W, Cell Signaling), β-actin (13E5, Cell Signaling), caspase-1 (Casper-1, AdipoGen), ASC (AL177, AdipoGen), IL-1β (B122, Santa Cruz Biotechnology). To detect activated caspase-1 p20 and released IL-1β, soluble proteins from supernatants were precipitated with methanol/chloroform. Briefly, cell culture supernatants were precipitated by the addition of an equal volume of methanol and 0.25 volumes of chloroform, followed by vortexing and centrifugation for 10 min at 20,000 × g. The upper phase was discarded and 500 μl methanol was added to the interphase. This mixture was centrifuged for 10 min at 20,000 × g and the protein pellet was dried on a speed vacuum for 5 min, and resuspended in RIPA buffer containing protease/phosphatase inhibitors. Following incubation with peroxydase-conjugated secondary antibodies, membranes were revealed by WesternBright Sirius HRP substrate (Advansta) in the Azure 300 instrument (Azure Biosystems). Relative protein expression was calculated by densitometric analysis (ImageJ software). Ratios between integrated density values obtained for the target protein and β-actin were calculated. Fold changes were expressed using the control sample (calibrator), with control values of uninfected/unstimulated samples being set to 1.

### RNA extraction and quantitative real-time PCR

Primary and immortalized mouse microglia stimulated with LPS/Aβ or LPS/Nig, in the absence or presence of the parasites were washed with PBS and were lysed with Qiazol reagent (Qiagen). Total RNA was isolated by using the QIAamp RNA Blood Mini Kit (Qiagen) according to manufacturer recommendations. qRT-PCR was carried out in MicroAmp™ Optical 96-well PCR plates (Applied Biosystems) using the Power SYBR™ Green RNA-to-CT™ 1-Step Kit (Applied Biosystems) and 0.3 mM primers with a 7500 Real-Time PCR System (Applied Biosystems). Primer information for every target tested by RT-qPCR is detailed in Table 1. Additionally, for *tnfaip3* and *hprt*, predesigned TaqMan Assays (Thermofisher Scientific) were used in combination with the TaqMan™ Fast Advanced Master Mix (Applied Biosystems). Probes used are as follows: TaqMan gene expression assay – Hprt (Mm03024075_m1), TaqMan gene expression assay – Tnfaip3 (Mm00437121_m1). Normalization was performed using the geometric mean of *gapdh* or *hprt* quantities. For statistical analysis of gene expression levels, Cp values were first transformed into relative quantities (RQ) and normalized. Nonparametric Kruskal–Wallis tests were performed on Log transformed Normalized Relative Quantity values.

**Table 1:** Oligonucleotides used for gene target amplification by RT-qPCR

Target	FW	RV
*IL-1β*	TGCCACCTTTTGACAGTGATG	GCTGCGAGATTTGAAGCTGG
*Nlrp3*	AGCTGGGGTTGGTGAATTCC	GTTTACAGTCCGGGTGCAGA
*Pycard*	ACTGTGCTTAGAGACATGGGC	CACAAAGTGTCCTGTTCTGGC
*Casp1*	CCATGGCTGACAAGATCCTGA	GATCACATAGGTCCCGTGCC
*Gapdh*	TGCAGTGGCAAAGTGGAGAT	CGTGAGTGGAGTCATACTGGAA

### Acridine orange staining of lysosomes in live cells

Microglia were seeded in complete medium on Lab-Tek glass chamber slides (Nunc) at 5 × 10^4^ cells. LMP was assessed in microglia sequentially treated with LPS and Aβ (1–42) (5 μM) in the presence or the absence of *L. infantum* parasites for 24 h. In addition, uninfected and infected microglia were treated with the lysosomotropic agent LLOMe (0.5 mM) for 30 min, a potent inducer of LMP. After treatment, cells were washed twice in PBS and stained with 5 μg/ml acridine orange (AO) and 1 μg/ml 4′,6-diamidino-2-phenylindole (DAPI) for 15 min at 37 °C in a 5% CO_2_ atmosphere, followed by five washes in PBS. Live-cell imaging of microglia was performed under an epifluorescence Eclipse Ti2 microscope (Nikon) with a 40 × objective (NA 1.4).

### Immunofluorescence analysis (IFA)

Microglia were seeded in complete medium on Lab-Tek glass chamber slides (Nunc) at 5 × 10^4^ cells per well followed by infection or not, and stimulated with LPS and fibrillar Aβ (1–42), nigericin or LLOMe. Cells were washed twice in PBS and fixed in 4% PFA for 15 min at room temperature. Then, slides were blocked in PBS, 5% normal serum and 0.3% Triton™ X-100 for 30 min (blocking buffer), followed by overnight incubation with the primary antibody in antibody dilution buffer (PBS, 1% BSA and 0.3% Triton X-100). Cells were washed three times in PBS for 5 min and incubated with goat-anti mouse, goat-anti rabbit or goat-anti rat Alexa Fluor 488-/Cy3-/Alexa Fluor 555-conjugated secondary antibodies (1:500, Jackson Immunoresearch and Invitrogen) for 90 min, then washed three times in PBS for 5 min. Slides were mounted using Fluoroshield™ with DAPI mounting medium (Sigma). The following primary antibodies were used with respective concentrations: rabbit anti-mouse Iba-1 (1:200, Cell Signaling), rabbit anti-mouse NFκB p65 (1:200, Cell Signaling), rabbit anti-mouse PE-ASC/TMS1 (1:200, Cell Signaling), rabbit anti-mouse CD68 (1:200, Abcam), rabbit anti-mouse TNFAIP3/A20 (1:200, Abcam), rat anti-mouse Gal-3 (1:1000, Santa Cruz Biotechnology), rat anti-mouse F4/80 (1:200, BioLegend). Slides were observed under an epifluorescence Eclipse Ti2 microscope (Nikon) with a 100 × objective (NA 1.4), an EL6000 (Leica) as light excitation source and controlled by the Micro-Manager V1.4.22 software (NIH). Images were acquired using a PRIM95B (Photometrics) camera and analyzed with ImageJ software V2.0.0 (NIH).

### Intracellular ASC/NLRP3 protein detection with the Amnis FlowSight imaging flow cytometer

Microglial cells were seeded in 6-well plates at 10^6^ cells per well, followed by infection or not, and stimulated with fibrillar Aβ (1–42) or nigericin. After 24 h of stimulation and/or infection, cells were detached, washed once in PBS, and fixed in PFA 1% for 15 min followed by permeabilization in 0.1% saponin in PBS for 30 min. After washing, cell pellets were stained in 0.1% saponin in PBS containing 5 μl anti-ASC and anti-NLRP3 antibodies (anti-mouse PE-ASC/TMS1, Cell Signaling; anti-mouse FITC-NLRP3/NALP3, Novus Biologicals) and incubated for 1 h at room temperature. After incubation, the cells were washed twice with PBS by centrifugation and then resuspended in 50 μl of pre-chilled PBS. They were subsequently analyzed using the Amnis FlowSight Imaging Flow Cytometer (Luminex Corporation, Austin, TX), as previously described [70]. All sample and compensation analyses were conducted using the IDEAS software. The IDEAS image analysis tool allows for the quantification of cellular morphology and fluorescence in different cellular regions by establishing specific cellular masks and applying mathematical expressions based on image pixel data or masks (features). The analysis of NLRP3 expression was conducted using an internalization feature, which involved a mask representing the entire cell based on the brightfield (BF) image, along with an internal mask created by eroding the whole cell mask. The formation of ASC specks was detected using a consistent mask for internalization features, which distinguished between diffuse fluorescence and speck (spot) fluorescence within cells. A threshold mask was applied to categorize the entire population of ASC-positive cells into two groups: ASC-speck cells and ASC-diffuse cells. ASC-speck cells were identified by their smaller spot area and higher maximum pixel intensity, while ASC-diffuse cells exhibited a larger area with lower pixel intensity, as described elsewhere [65].

### Statistical analysis

Data were analysed either by one-way ANOVA, followed by post hoc analysis where appropriate, or by two-tailed, unpaired Student’s t-test if not indicated otherwise, using Graph Pad Prism V10.2.0 for Mac OS. Statistical details are given in the respective figure legends. Standardization of fluorescent signals was carried out by parallel setting of raw integrated density signals in all the images to be compared in ImageJ V2.0.0 (NIH). For clarity purposes, the brightness and contrast of several pictures were adjusted after their analysis in accordance with editorial policies. Statistical analyses and plots were performed with Prism V10.2.0 (GraphPad).

## Supplementary Information

Additional file 1: Figure S1. Immortalized microglia express markers of primary microglia. A, B) Immunofluorescence images of primary microglia (A) and immortalized microglia (B) stained with anti-Iba-1 (red), anti-F4/80 (green) and anti-CD68 (magenta) antibodies. DNA staining with DAPI. Scale bars represent 10 µm. C) Normalized fluorescence images of PFA-fixed immortalized microglia infected or not with *L. infantum* and stained with an anti-Iba-1 antibody (green). DNA content was stained with DAPI. Scale bars are 10 µm. D) Quantification of the fluorescent signal of Iba-1 from images in C). The number of cells considered for quantification (n) is indicated above the graph. Statistical analysis: two-tailed, unpaired t test (****p<0.0001). E) Western blot analyses and relative quantification of Iba-1 expression in microglial cell lysates (iMG) in the presence or absence of *L. infantum*. Beta actin was used as a loading control protein. Data (mean ± SD) are representative of two separate experiments.

Additional file 2: Figure S2. The production of NLRP3-associated pro-inflammatory mediators is parasite-dependent. A, B, C, D) Detection by ELISA of secreted IL-1β (A), IL-18 (B), and TNF-α (C) cytokines, and nitric oxide by the Griess reaction (D). All pro-inflammatory mediators were quantified in the supernatants of iMG left untreated (Ctrl), or following LPS/Aβ and LPS/Nig stimulation in the absence or the presence of *L. infantum* parasites. Increasing cell:parasite ratios (1:5, 1:10 and 1:20) were used. Results represent the mean ± SD of two independent experiments; one-way ANOVA with Tukey’s multiple comparisons test (**p<0.01;***p<0.001; ****p<0.0001; ns, not significant).

Additional file 3: Figure S3. Inhibiting parasite phagocytosis blocks the *L. infantum*-mediated anti-inflammatory effect. A) ELISA of the release of IL-1β into supernatants of immortalized microglia treated with cytochalasin D (CytoD) before stimulation with LPS/Aβ, or after LPS/Aβ treatment followed by *L. infantum *infection. B) Transcriptional modulation of *proIL-1β* as assessed by qRT-PCR in the presence or absence of CytoD. The expression fold change is indicated using uninfected and unstimulated iMG as a calibrator. As a positive control for IL-1β inhibition, iMG were pretreated for 1 h with the caspase-1-specific inhibitor Ac-YVAD-cmk. C) Representative live-cell images of CytoD-treated iMG showing non phagocytosed and living *PpytdT+Li* parasites. Scale bar 10 µm. Results represent the mean ± SD of two independent experiments. Statistical differences according to one-way ANOVA and Tukey’s comparison tests (*p<0.05; ***p<0.0005;****p<0.0001; ns, not significant).

Additional file 4: Figure S4. The NLRP3 inflammasome priming step is inhibited by *L. infantum* in Aβ-stimulated immortalized microglia. A) Immunofluorescence images of PFA-fixed iMG stimulated or not with LPS/Aβ in the presence or the absence of the parasites and stained with an antibody anti-NF-κB p65 (RelA) (green). DNA was stained with DAPI (blue). White arrowheads show the parasite’s DNA within microglia. Scale bars represent 10 µm. B) Relative quantitation by WB analysis of NF-κB RelA in total protein extracts of uninfected and infected iMG stimulated with LPS/Aβ and LPS/Nig for 24 h. Beta-actin was used as a control protein. C, D) Transcriptional modulation of *nlrp3* (C) and *proIL-1β* (D) in iMG after 24 hours of infection and/or stimulation by qRT-PCR. Results represent the mean ± SD of three independent experiments (*n*=3) with two technical replicates per assay (*n*=6). Statistical differences according to one-way ANOVA and Tukey’s comparison tests (*p<0.05;**p<0.01; ***p<0.001; ****p<0.0001; ns, not significant).

Additional file 5: Figure S5. *L. infantum* prevents the formation of ASC specks in Aβ-activated immortalized microglia. A, B). Representative images of ASC speck detection using Aβ-stimulated iMG (positive cells) obtained with the IDEAS software of the Amnis FlowSight Imaging Flow Cytometer by applying a consistent internalization feature mask (A), to distinguish between ASC-speck (spot) and ASC-diffuse fluorescence within cells (B). C) Selected images of unstimulated and uninfected, infected, Aβ-activated, and infected Aβ-stimulated microglia: brightfield (BF) images allow observation of cell shape and density; ASC-PE red fluorescent pictures highlight the ASC protein specifically using a fluorescent tag (phycoerythrin, PE), indicating whether the cells are ASC-speck positive or ASC-diffuse; merged images combine the BF view with the ASC-PE fluorescence, providing a clearer visual context of the presence or absence of ASC specks within the cells. D) Representative images of ASC/NLRP3 co-localization in microglial cells stained with antibodies recognizing ASC (PE-labeled ASC) and NLRP3 (FITC-labeled NLRP3), and further analyzed using the IDEAS software of the Amnis FlowSight. ASC specks were not observed in unstimulated and infected cells, whereas ASC puncta were present in Aβ-stimulated microglia; *L. infantum* infection in Aβ-stimulated microglia resulted in diffuse ASC, indicating the suppression of NLRP3 activation. E) Percentage of ASC-speck positive microglia. Data are from three independent experiments and expressed as the mean ± SD. Statistical significance according to one-way ANOVA and Tukey’s comparison tests (*p<0.008; **p<0.004).

Additional file 6.

## Data Availability

All data generated or analysed during this study are included in this published article (and its Supplementary information files).

## Abbreviations

AD: Alzheimer’s disease
Aβ: Amyloid-beta peptide
NLRP3: NOD-, LRR- and pyrin domain-containing protein 3
IL-1β: Interleukin-1 beta
IL-18: Interleukin-18
TNF-α: Tumor necrosis factor α
NF-κB: Nuclear factor kappa-light-chain-enhancer of activated B cells
ASC: Apoptosis-associated speck-like protein containing a CARD
ROS: Reactive oxygen species
DAMP: Damage-associated molecular pattern
MS: Multiple sclerosis
ASD: Autism spectrum disorder
ALS: Amyotrophic lateral sclerosis
pMG: Primary microglia
iMG: Immortalized microglia
hpi: Hours post infection
MTT: 3-(4,5-Dimethylthiazol-2-yl)-2,5-diphenyltetrazolium bromide
LDH: Lactate dehydrogenase
NO: Nitric oxide
Iba-1: Ionized calcium-binding adaptor molecule 1
LPS: Lipopolysaccharide
iNOS: Inducible nitric oxide synthase
Nig: Nigericin
CytoD: Cytochalasin D
A20/TNFAIP3: TNF-α-induced protein 3
WT: Wild-type
KO: Knockout
LMP: Lysosomal membrane permeabilization
Gal-3: Galectin 3
LLOMe: L-Leucyl-L-Leucine methyl ester
CNS: Central nervous system
