# Supplementary figures and images for: Immune subversion by Leishmania infantum parasites suppresses NLRP3-driven inflammatory responses in amyloid-β-activated microglia
Source: J Neuroinflammation. 2025 Oct 29;22:252. doi: 10.1186/s12974-025-03574-5 (PMC12573824; doi:10.1186/s12974-025-03574-5)

# Figure S1

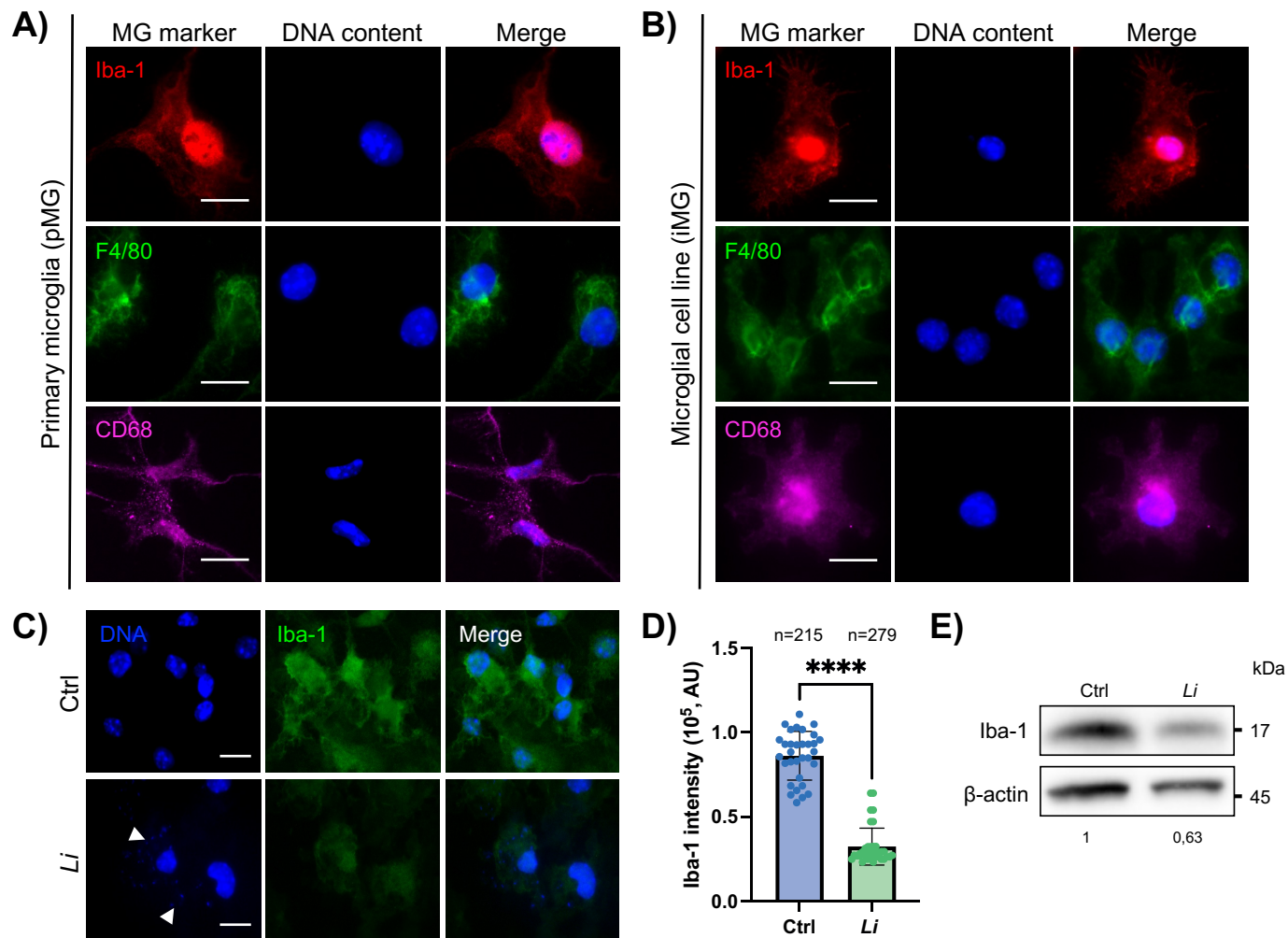

Supplement: Supplementary file 1 — Additional file 1: Figure S1. Immortalized microglia express markers of primary microglia. A, B) Immunofluorescence images of primary microglia (A) and immortalized microglia (B) stained with anti-Iba-1 (red), anti-F4/80 (green) and anti-CD68 (magenta) antibodies. DNA staining with DAPI. Scale bars represent 10 µm. C) Normalized fluorescence images of PFA-fixed immortalized microglia infected or not with L. infantum and stained with an anti-Iba-1 antibody (green). DNA content was stained with DAPI. Scale bars are 10 µm. D) Quantification of the fluorescent signal of Iba-1 from images in C). The number of cells considered for quantification (n) is indicated above the graph. Statistical analysis: two-tailed, unpaired t test (****p<0.0001). E) Western blot analyses and relative quantification of Iba-1 expression in microglial cell lysates (iMG) in the presence or absence of L. infantum. Beta actin was used as a loading control protein. Data (mean ± SD) are representative of two separate experiments. [file 12974_2025_3574_MOESM1_ESM.pdf]

Figure S2

A)

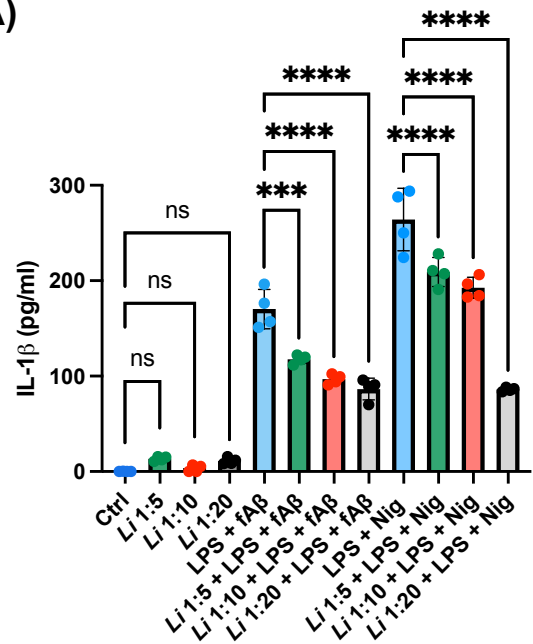

B)

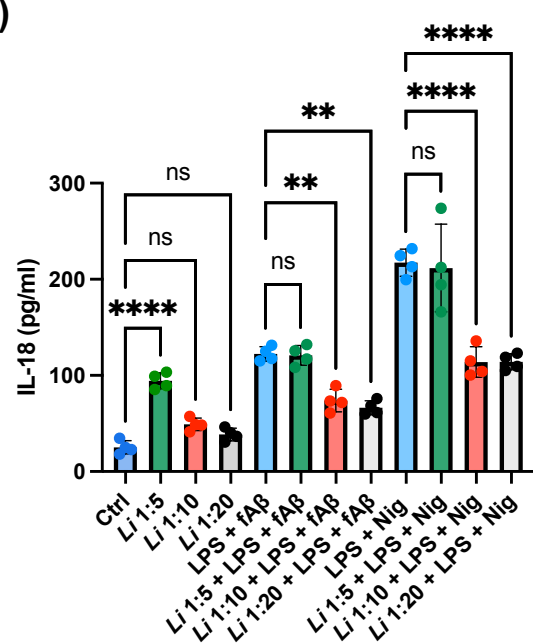

C)

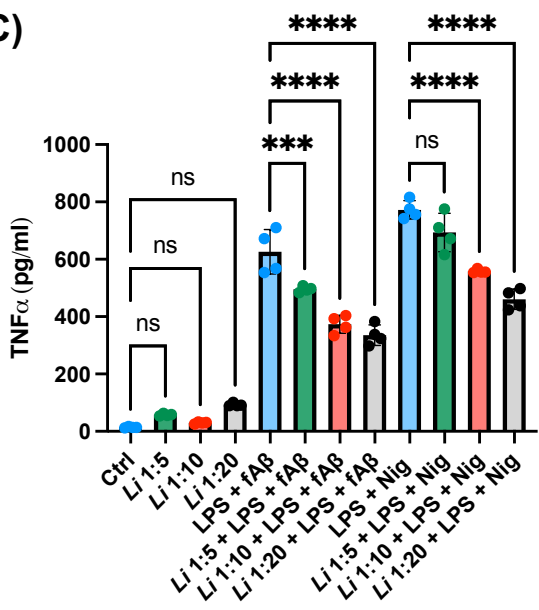

D)

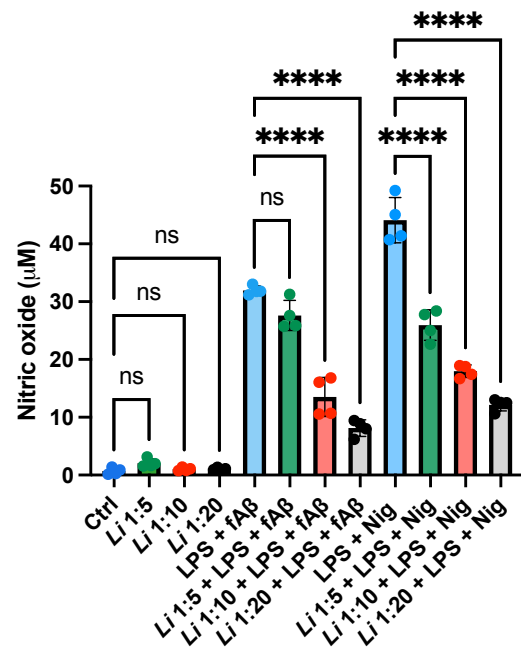

Supplement: Supplementary file 2 — Additional file 2: Figure S2. The production of NLRP3-associated pro-inflammatory mediators is parasite-dependent. A, B, C, D) Detection by ELISA of secreted IL-1β (A), IL-18 (B), and TNF-α (C) cytokines, and nitric oxide by the Griess reaction (D). All pro-inflammatory mediators were quantified in the supernatants of iMG left untreated (Ctrl), or following LPS/Aβ and LPS/Nig stimulation in the absence or the presence of L. infantum parasites. Increasing cell:parasite ratios (1:5, 1:10 and 1:20) were used. Results represent the mean ± SD of two independent experiments; one-way ANOVA with Tukey’s multiple comparisons test (**p<0.01;***p<0.001; ****p<0.0001; ns, not significant). [file 12974_2025_3574_MOESM2_ESM.pdf]

# Figure S3

A)

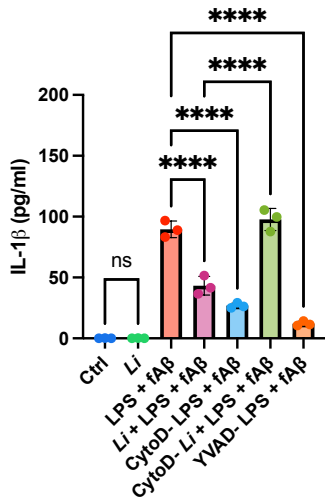

B)

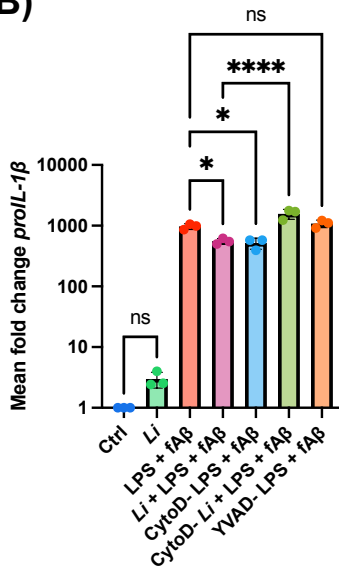

C)

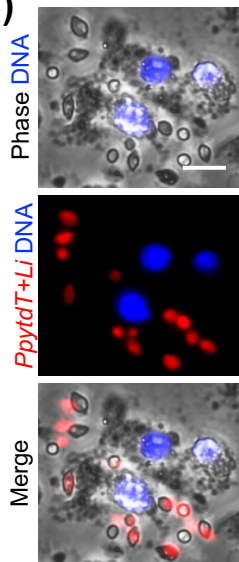

Supplement: Supplementary file 3 — Additional file 3: Figure S3. Inhibiting parasite phagocytosis blocks the L. infantum-mediated anti-inflammatory effect. A) ELISA of the release of IL-1β into supernatants of immortalized microglia treated with cytochalasin D (CytoD) before stimulation with LPS/Aβ, or after LPS/Aβ treatment followed by L. infantum infection. B) Transcriptional modulation of proIL-1β as assessed by qRT-PCR in the presence or absence of CytoD. The expression fold change is indicated using uninfected and unstimulated iMG as a calibrator. As a positive control for IL-1β inhibition, iMG were pretreated for 1 h with the caspase-1-specific inhibitor Ac-YVAD-cmk. C) Representative live-cell images of CytoD-treated iMG showing non phagocytosed and living PpytdT+Li parasites. Scale bar 10 µm. Results represent the mean ± SD of two independent experiments. Statistical differences according to one-way ANOVA and Tukey’s comparison tests (*p<0.05; ***p<0.0005;****p<0.0001; ns, not significant). [file 12974_2025_3574_MOESM3_ESM.pdf]

# Figure S4

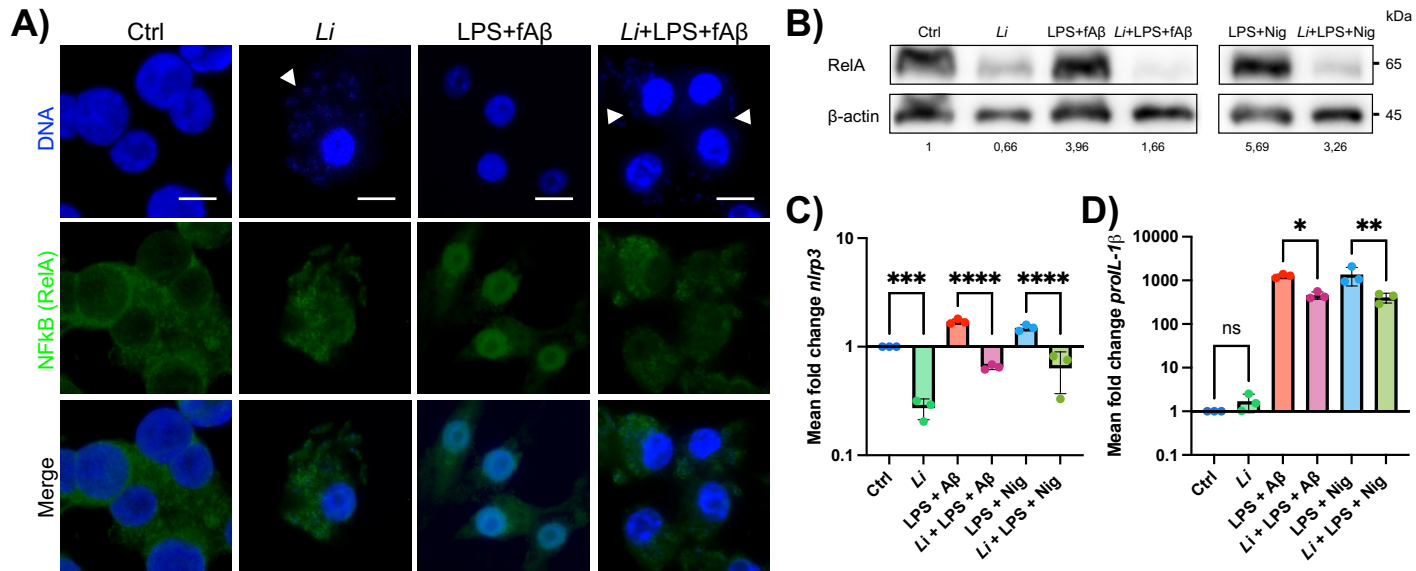

Supplement: Supplementary file 4 — Additional file 4: Figure S4. The NLRP3 inflammasome priming step is inhibited by L. infantum in Aβ-stimulated immortalized microglia. A) Immunofluorescence images of PFA-fixed iMG stimulated or not with LPS/Aβ in the presence or the absence of the parasites and stained with an antibody anti-NF-κB p65 (RelA) (green). DNA was stained with DAPI (blue). White arrowheads show the parasite’s DNA within microglia. Scale bars represent 10 µm. B) Relative quantitation by WB analysis of NF-κB RelA in total protein extracts of uninfected and infected iMG stimulated with LPS/Aβ and LPS/Nig for 24 h. Beta-actin was used as a control protein. C, D) Transcriptional modulation of nlrp3 (C) and proIL-1β (D) in iMG after 24 hours of infection and/or stimulation by qRT-PCR. Results represent the mean ± SD of three independent experiments (n=3) with two technical replicates per assay (n=6). Statistical differences according to one-way ANOVA and Tukey’s comparison tests (*p<0.05;**p<0.01; ***p<0.001; ****p<0.0001; ns, not significant). [file 12974_2025_3574_MOESM4_ESM.pdf]

# Figure S5

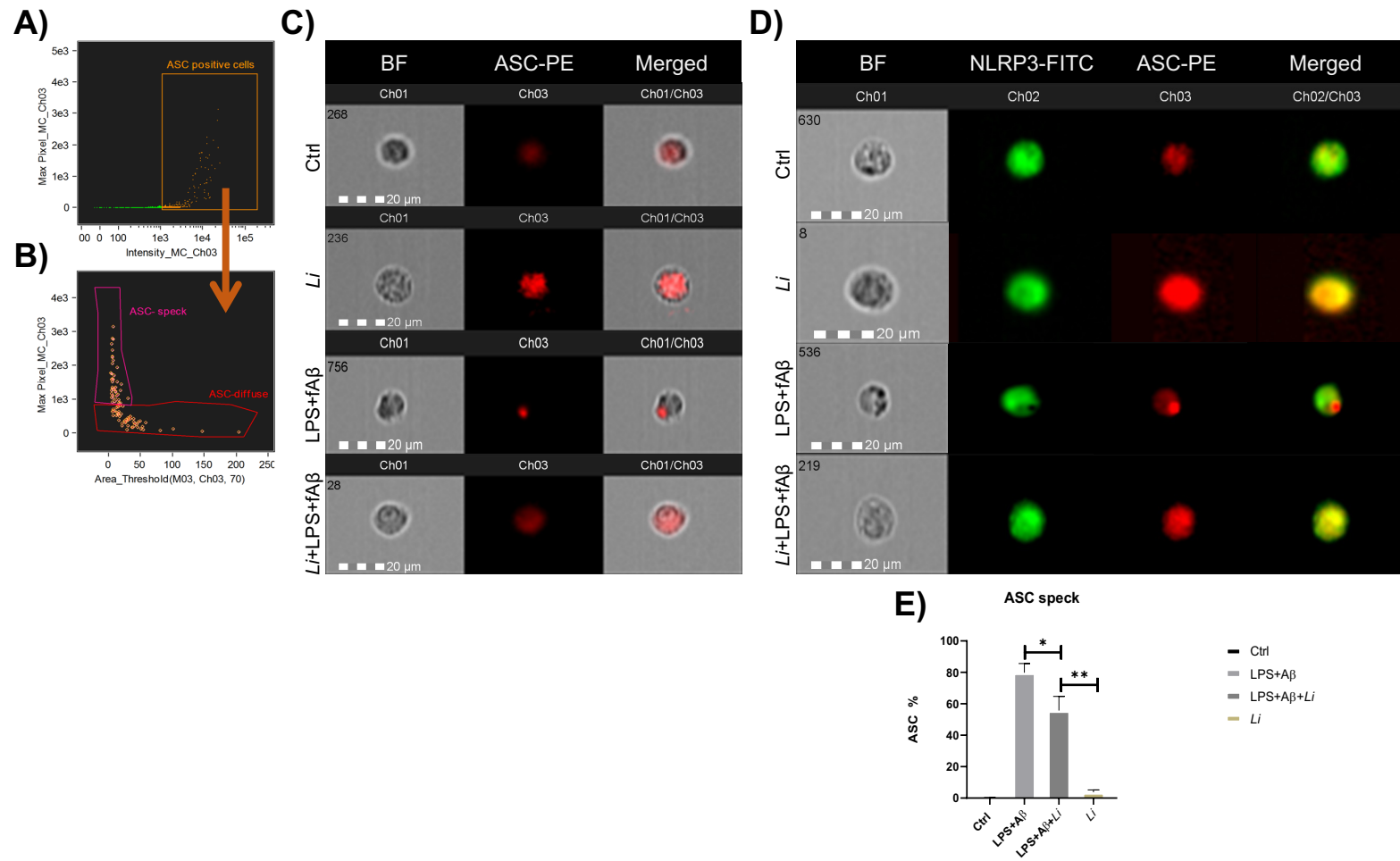

Supplement: Supplementary file 5 — Additional file 5: Figure S5. L. infantum prevents the formation of ASC specks in Aβ-activated immortalized microglia. A, B). Representative images of ASC speck detection using Aβ-stimulated iMG (positive cells) obtained with the IDEAS software of the Amnis FlowSight Imaging Flow Cytometer by applying a consistent internalization feature mask (A), to distinguish between ASC-speck (spot) and ASC-diffuse fluorescence within cells (B). C) Selected images of unstimulated and uninfected, infected, Aβ-activated, and infected Aβ-stimulated microglia: brightfield (BF) images allow observation of cell shape and density; ASC-PE red fluorescent pictures highlight the ASC protein specifically using a fluorescent tag (phycoerythrin, PE), indicating whether the cells are ASC-speck positive or ASC-diffuse; merged images combine the BF view with the ASC-PE fluorescence, providing a clearer visual context of the presence or absence of ASC specks within the cells. D) Representative images of ASC/NLRP3 co-localization in microglial cells stained with antibodies recognizing ASC (PE-labeled ASC) and NLRP3 (FITC-labeled NLRP3), and further analyzed using the IDEAS software of the Amnis FlowSight. ASC specks were not observed in unstimulated and infected cells, whereas ASC puncta were present in Aβ-stimulated microglia; L. infantum infection in Aβ-stimulated microglia resulted in diffuse ASC, indicating the suppression of NLRP3 activation. E) Percentage of ASC-speck positive microglia. Data are from three independent experiments and expressed as the mean ± SD. Statistical significance according to one-way ANOVA and Tukey’s comparison tests (*p<0.008; **p<0.004). [file 12974_2025_3574_MOESM5_ESM.pdf]
