# Supplementary material for: Immune subversion by Leishmania infantum parasites suppresses NLRP3-driven inflammatory responses in amyloid-β-activated microglia
Source: J Neuroinflammation. 2025 Oct 29;22:252. doi: 10.1186/s12974-025-03574-5 (PMC12573824; doi:10.1186/s12974-025-03574-5)
Supplement: Supplementary file 6 — Additional file 6. [file 12974_2025_3574_MOESM6_ESM.pdf]

Figure 2

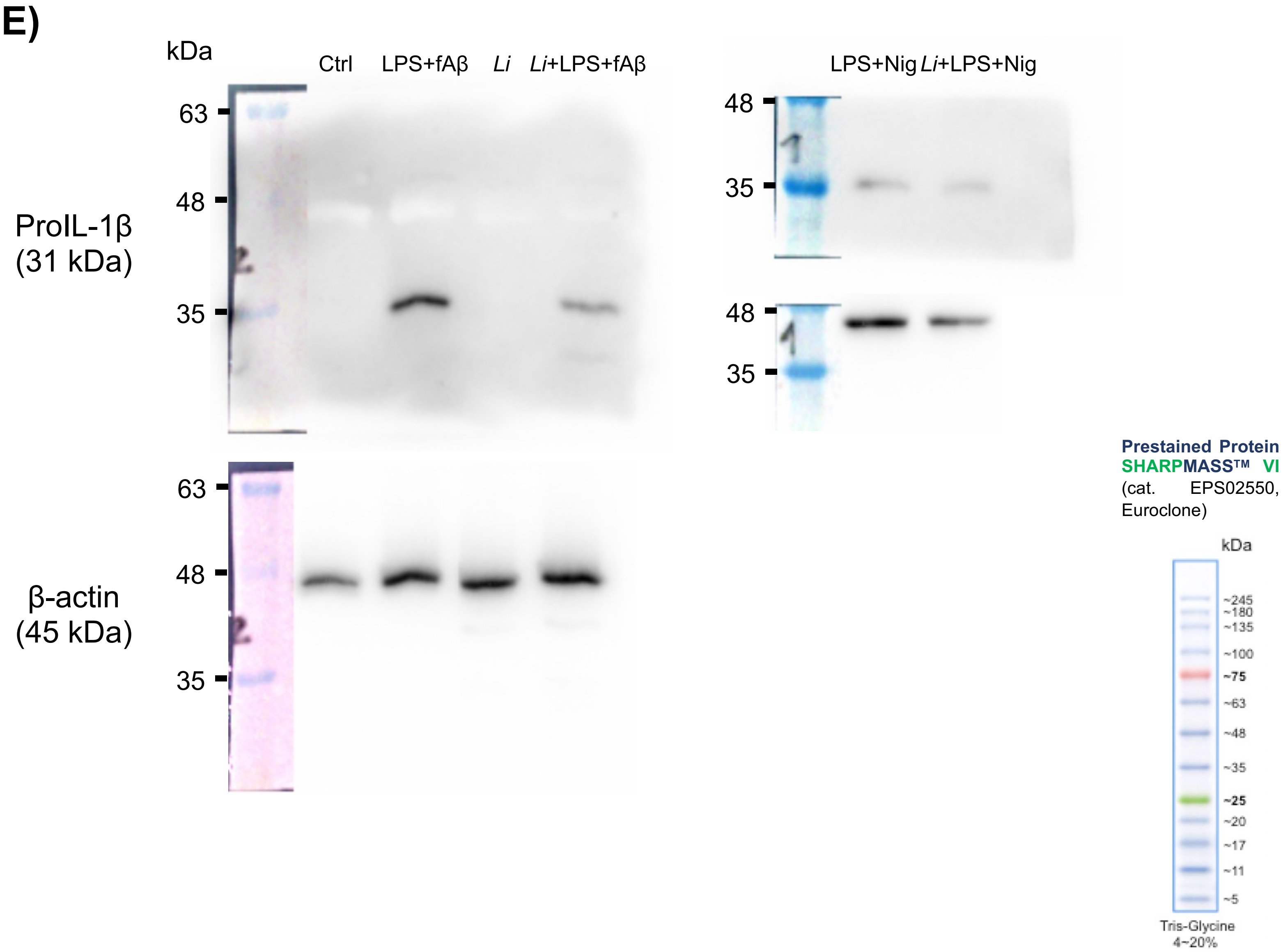
E)
kDa
Ctrl
LPS+fAβ
Li
Li+LPS+fAβ
LPS+Nig
Li+LPS+Nig
63
48
35
48
35
48
35
ProIL-1β
(31 kDa)
63
48
35
β-actin
(45 kDa)
Prestained Protein
SHARPMASS™ VI
(cat. EPS02550,
Euroclone)
kDa
~245
~180
~135
~100
~75
~63
~48
~35
~25
~20
~17
~11
~5
Tris-Glycine
4~20%

Figure 2

E)

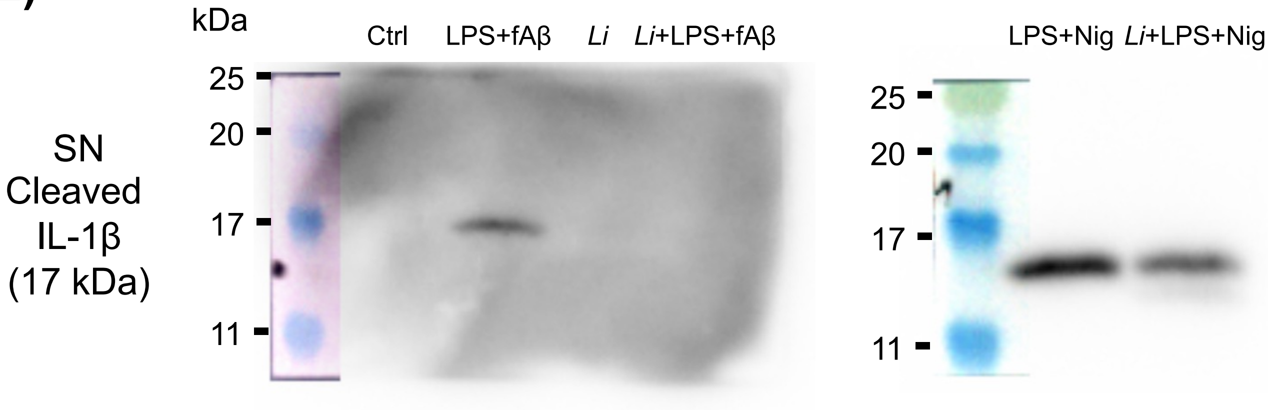

Prestained Protein SHARPMASS™ VI (cat. EPS02550, Euroclone)

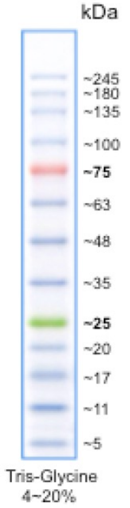

Figure 2

F)

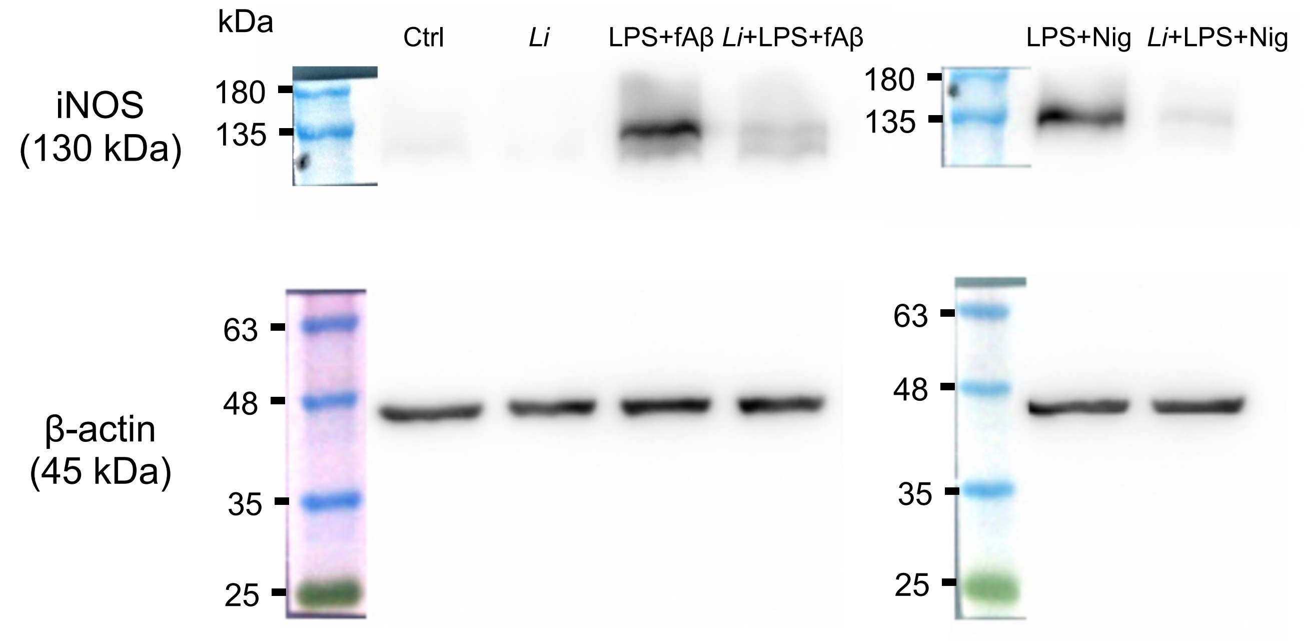

kDa
Ctrl
Li
LPS+fAβ
Li+LPS+fAβ
LPS+Nig
Li+LPS+Nig
iNOS
(130 kDa)
180
135
180
135
β-actin
(45 kDa)
63
48
35
25
63
48
35
25

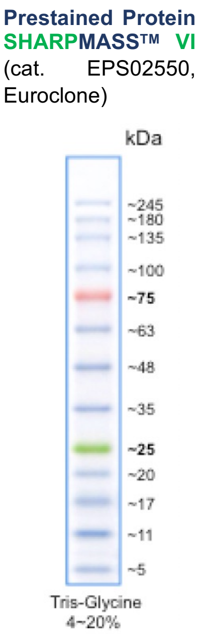

Prestained Protein SHARPMASS™ VI (cat. EPS02550, Euroclone)
kDa
~245
~180
~135
~100
~75
~63
~48
~35
~25
~20
~17
~11
~5
Tris-Glycine 4~20%

Figure 3

C)

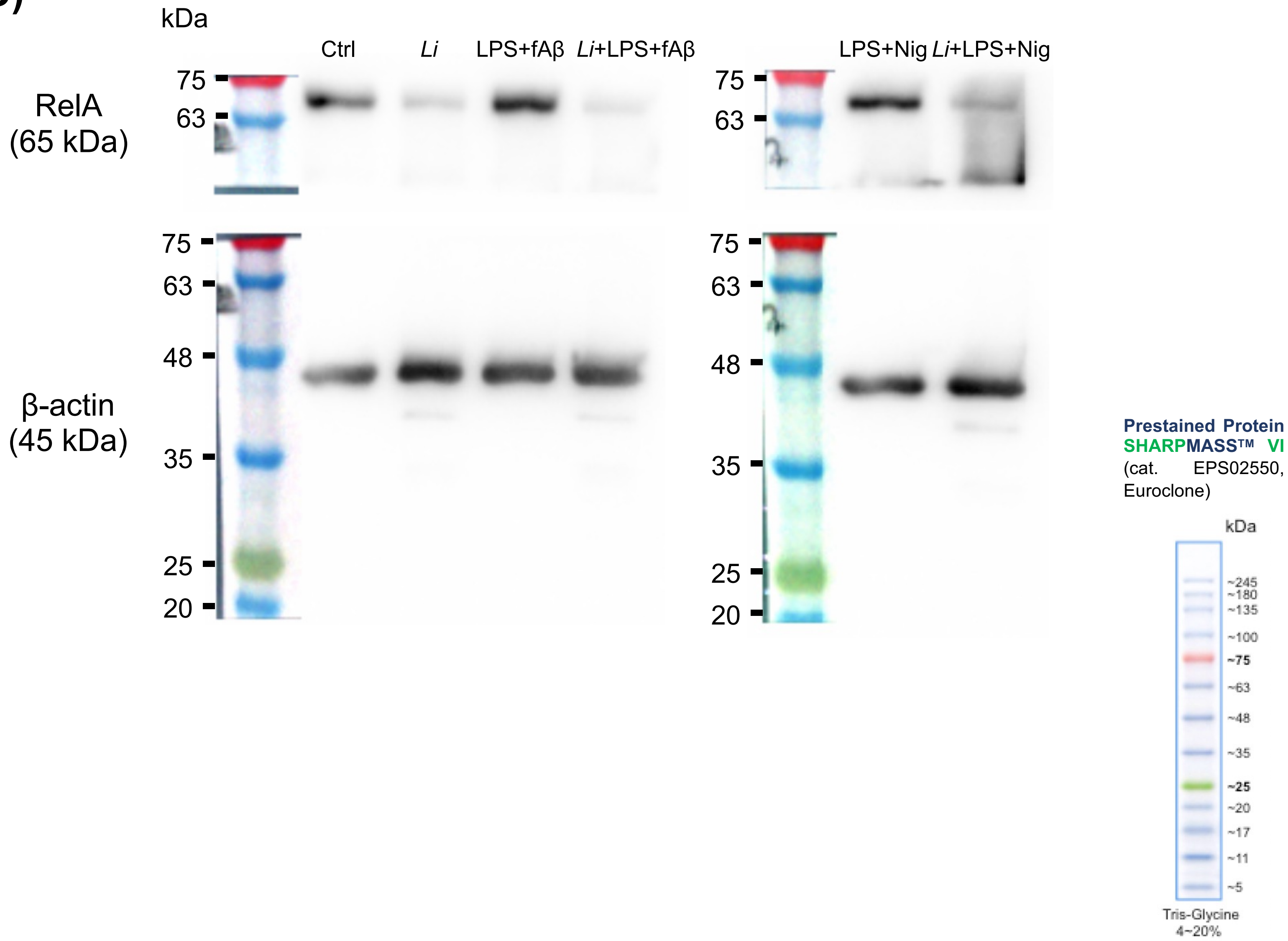
kDa
Ctrl
Li
LPS+fAβ
Li+LPS+fAβ
LPS+Nig
Li+LPS+Nig
RelA
(65 kDa)
β-actin
(45 kDa)
75
63
48
35
25
20
Prestained Protein SHARPMASS™ VI (cat. EPS02550, Euroclone)
kDa
~245
~180
~135
~100
~75
~63
~48
~35
~25
~20
~17
~11
~5
Tris-Glycine 4~20%

Figure 3

G)

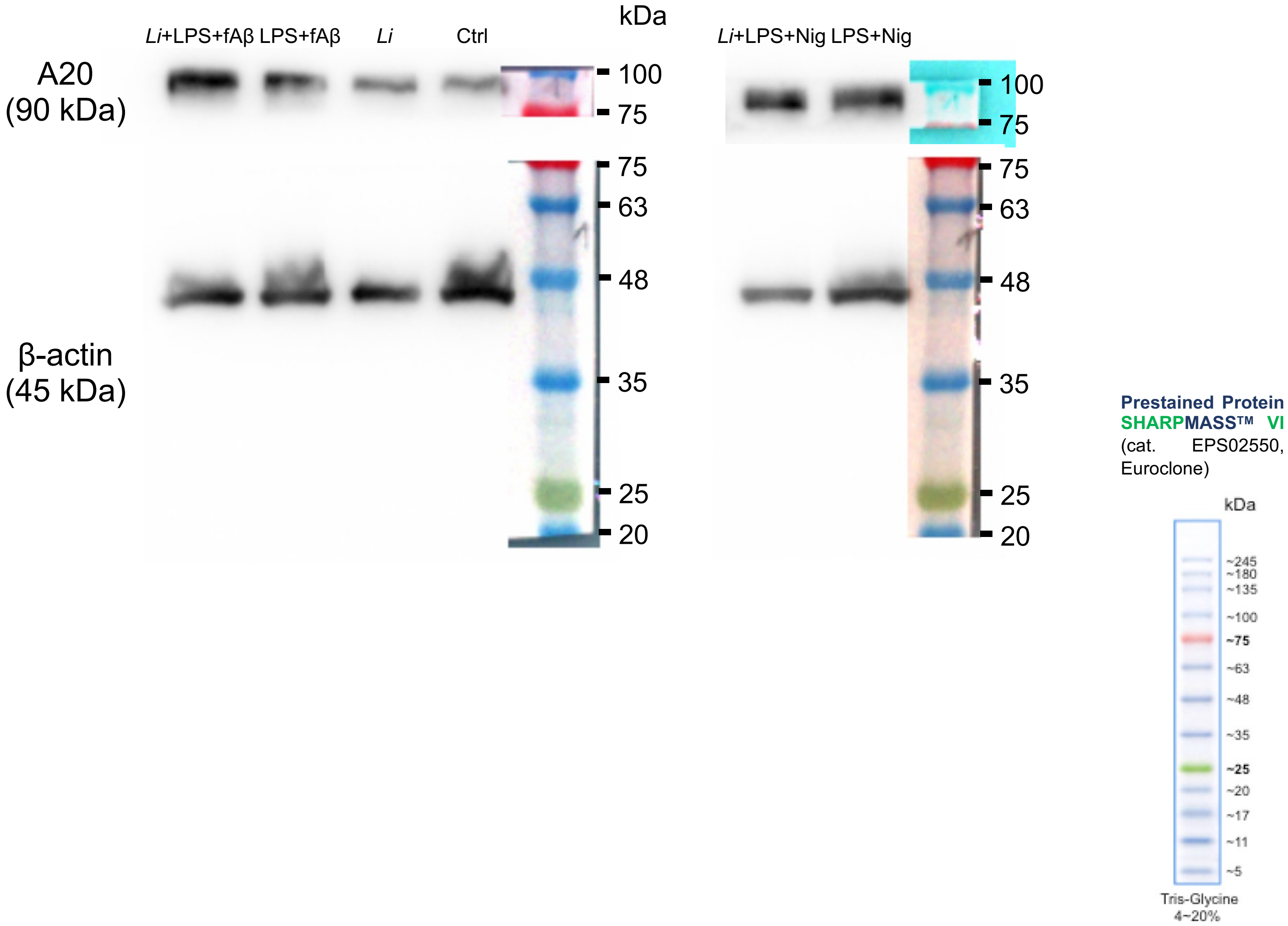

kDa
Li+LPS+fAβ LPS+fAβ Li Ctrl
A20
(90 kDa)
100
75
β-actin
(45 kDa)
75
63
48
35
25
20
Li+LPS+Nig LPS+Nig
100
75
75
63
48
35
25
20
Prestained Protein
SHARPMASS™ VI
(cat. EPS02550,
Euroclone)
kDa
~245
~180
~135
~100
~75
~63
~48
~35
~25
~20
~17
~11
~5
Tris-Glycine
4~20%

Figure 4

B)

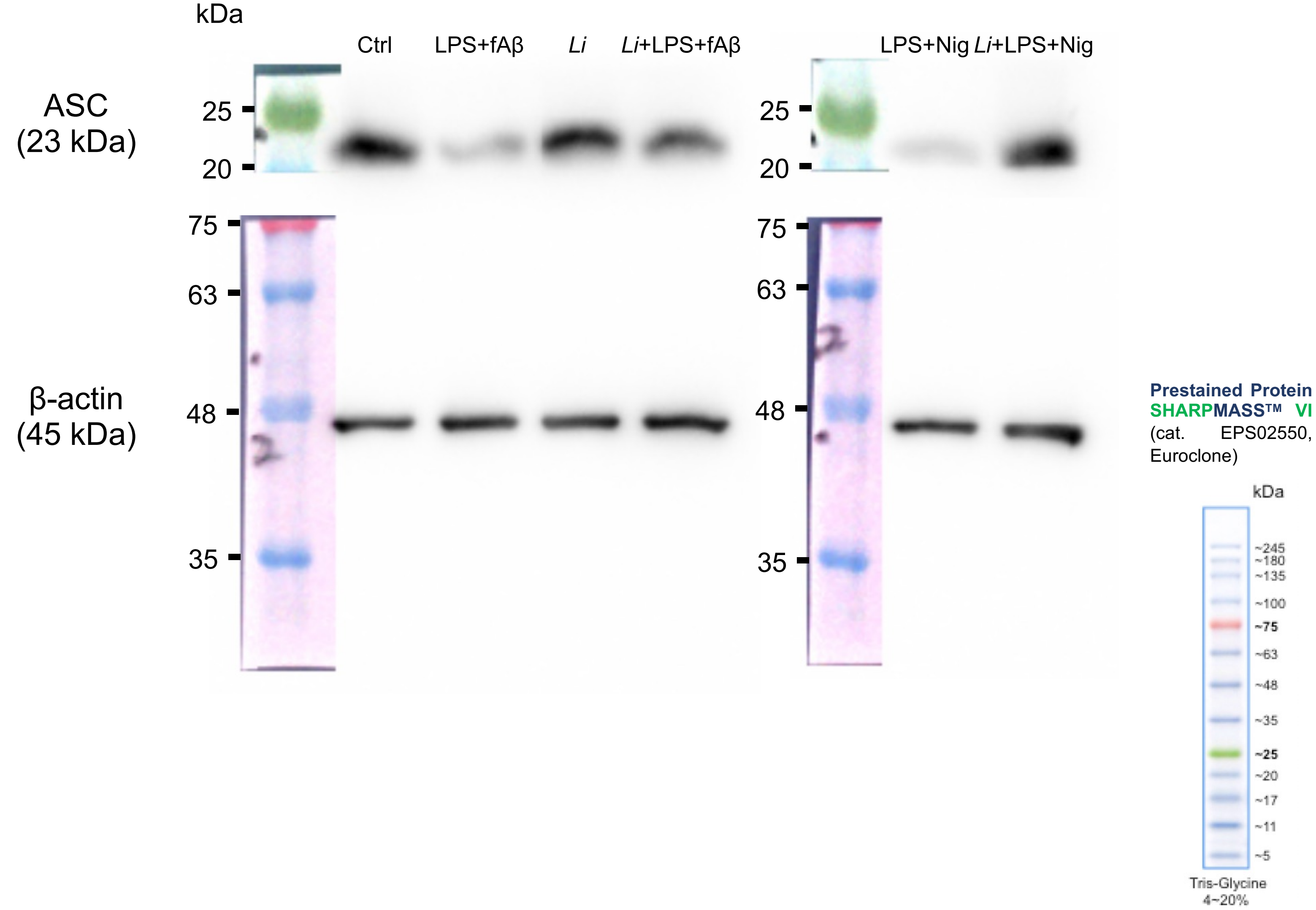

kDa
Ctrl
LPS+fAβ
Li
Li+LPS+fAβ
LPS+Nig
Li+LPS+Nig
ASC
(23 kDa)
25
20
25
20
75
63
48
35
75
63
48
35
β-actin
(45 kDa)
Prestained Protein
SHARPMASS™ VI
(cat. EPS02550,
Euroclone)
kDa
~245
~180
~135
~100
~75
~63
~48
~35
~25
~20
~17
~11
~5
Tris-Glycine
4~20%

Figure 4

G)

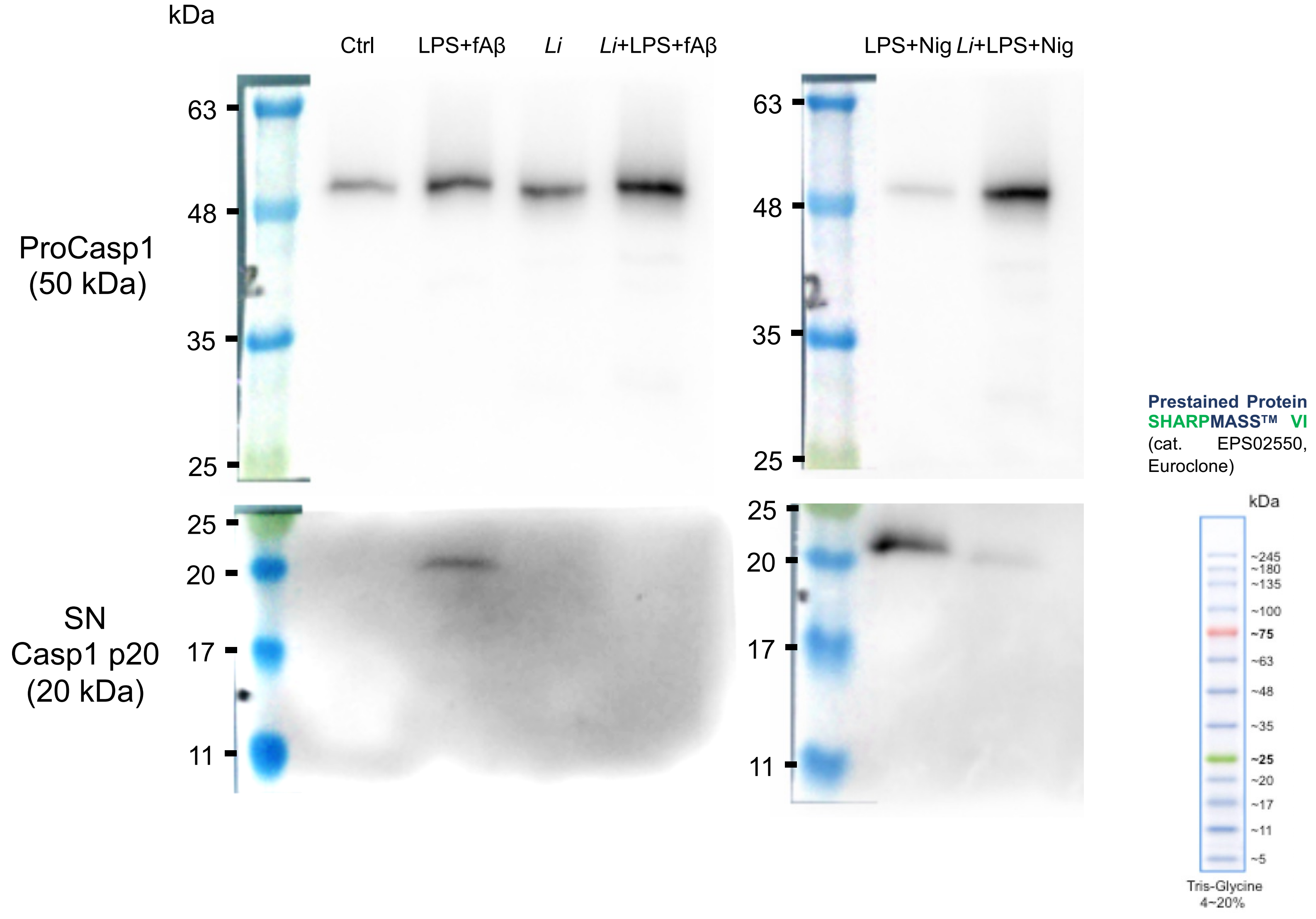

kDa
Ctrl
LPS+fAβ
Li
Li+LPS+fAβ
LPS+Nig
Li+LPS+Nig
63
48
35
25
20
17
11
ProCasp1 (50 kDa)
SN Casp1 p20 (20 kDa)
Prestained Protein SHARPMASS™ VI (cat. EPS02550, Euroclone)
~245
~180
~135
~100
~75
~63
~48
~35
~25
~20
~17
~11
~5
Tris-Glycine 4~20%

Figure S1

E)

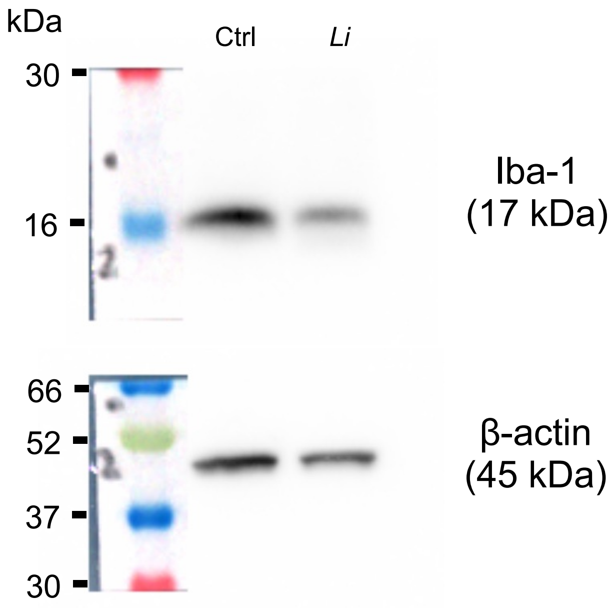

Opti-Protein Ultra marker (cat. G623, ABM)

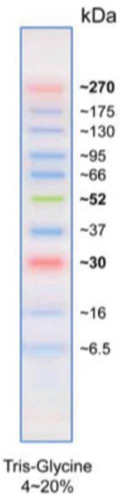

Figure S4

B)

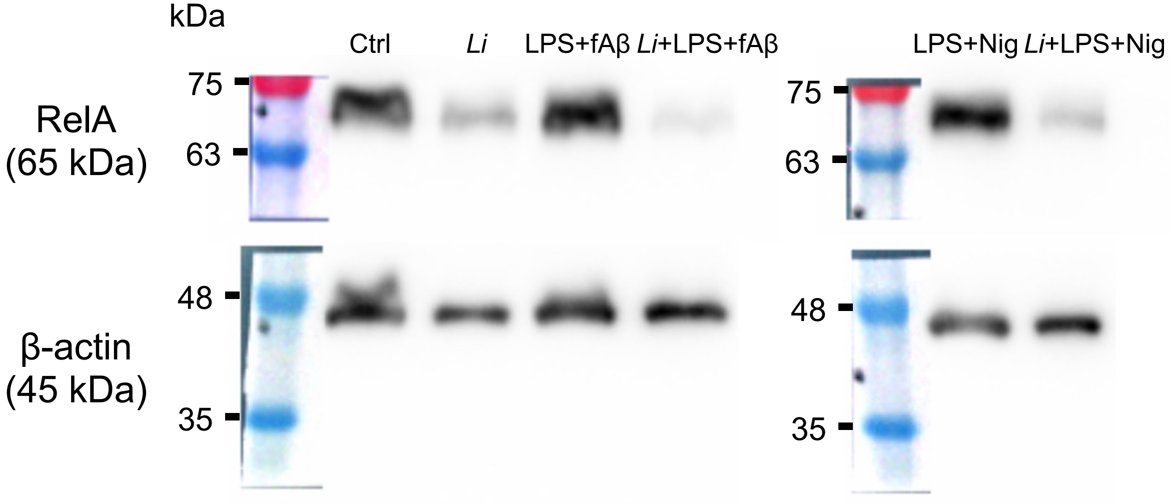

kDa
Ctrl
Li
LPS+fAβ
Li+LPS+fAβ
LPS+Nig
Li+LPS+Nig
RelA
(65 kDa)
75
63
β-actin
(45 kDa)
48
35

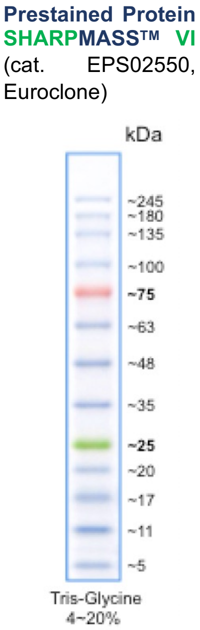

Prestained Protein SHARPMASS™ VI (cat. EPS02550, Euroclone)
kDa
~245
~180
~135
~100
~75
~63
~48
~35
~25
~20
~17
~11
~5
Tris-Glycine 4~20%
